# Population-based screening in a municipality after a primary school outbreak of the SARS-CoV-2 Alpha variant, the Netherlands, December 2020–February 2021

**DOI:** 10.1371/journal.pone.0276696

**Published:** 2022-10-27

**Authors:** Janko van Beek, Gwen Teesing, Bas B. Oude Munnink, Abraham Meima, Henrike J. Vriend, Jessica Elzakkers, Miranda de Graaf, Jeroen Langeveld, Gert-Jan Medema, Richard Molenkamp, Helene Voeten, Ewout Fanoy, Marion Koopmans, Outbreak investigation and national genomic surveillance teams

**Affiliations:** 1 Department of Viroscience, Erasmus MC, University Medical Center Rotterdam, Rotterdam, The Netherlands; 2 Department of Infectious Disease Control, Public Health Service Rotterdam-Rijnmond, Rotterdam, The Netherlands; 3 The Netherlands Organization for Health Research and Development (ZonMw), The Hague, The Netherlands; 4 KWR Water Research Institute, Nieuwegein, The Netherlands; 5 Partners4UrbanWater, Nijmegen, The Netherlands; 6 Sanitary Engineering, Delft University of Technology, Delft, The Netherlands; 7 Department of Public Health, Erasmus MC, University Medical Center Rotterdam, Rotterdam, The Netherlands; Health Directorate, LUXEMBOURG

## Abstract

An outbreak of SARS-CoV-2 Alpha variant (Pango lineage B.1.1.7) was detected at a primary school (School X) in Lansingerland, the Netherlands, in December 2020. The outbreak was studied retrospectively, and population-based screening was used to assess the extent of virus circulation and decelerate transmission. Cases were SARS-CoV-2 laboratory confirmed and were residents of Lansingerland (November 16^th^ 2020 until February 22^th^ 2021), or had an epidemiological link with School X or neighbouring schools. The SARS-CoV-2 variant was determined using variant PCR or whole genome sequencing. A questionnaire primarily assessed clinical symptoms. A total of 77 Alpha variant cases were found with an epidemiological link to School X, 16 Alpha variant cases linked to the neighbouring schools, and 146 Alpha variant cases among residents of Lansingerland without a link to the schools. The mean number of self-reported symptoms was not significantly different among Alpha variant infected individuals compared to non-Alpha infected individuals. The secondary attack rate (SAR) among Alpha variant exposed individuals in households was 52% higher compared to non-Alpha variant exposed individuals (p = 0.010), with the mean household age, and mean number of children and adults per household as confounders. Sequence analysis of 60 Alpha variant sequences obtained from cases confirmed virus transmission between School X and neighbouring schools, and showed that multiple introductions of the Alpha variant had already taken place in Lansingerland at the time of the study. The alpha variant caused a large outbreak at both locations of School X, and subsequently spread to neighbouring schools, and households. Population-based screening (together with other public health measures) nearly stopped transmission of the outbreak strain, but did not prevent variant replacement in the Lansingerland municipality.

## Background

The respiratory illness COVID-19 is caused by the severe acute respiratory syndrome coronavirus 2 (SARS-CoV-2), which emerged in Wuhan, China, late 2019 [1]. SARS-CoV-2 rapidly spread globally and caused a major public health and economic impact. Meta-analysis of transmission studies during the early phase of the pandemic suggested that children and adolescents had a reduced susceptibility to SARS-CoV-2 compared to adults, and contributed less to the spread of the pandemic, compared with for instance influenza pandemics [2, 3].

The variant of concern (VOC) SARS-CoV-2 Alpha (Pango lineage B.1.1.7) was first detected in November, in samples collected in September 2020 from cases in the United Kingdom (UK) [4]. This variant became the predominant strain in the UK in December 2020, only weeks after it had first been identified. The Alpha variant genome is characterised by 17 amino acid mutations, including a N501Y mutation in the receptor binding pocket of the Spike (S) protein, compared to the Wuhan wildtype (with D614G) strains [5]. Initial epidemiological studies suggested an overrepresentation of Alpha variant cases among the age group 0–19 years of age, and a higher reproduction number for Alpha variant compared to pre-existing variants [6–8].

Following the public health alert from the UK, increased surveillance was done in the Netherlands, including testing of travellers from the UK and sequencing of a random sample of positives. This resulted in the first detection of an Alpha variant infection in the Netherlands in December 2020, and contact tracing showed a link with the first Dutch Alpha variant case and an outbreak at a primary school (School X) in the municipality Lansingerland. We studied the outbreak retrospectively and prospectively in order to get a better understanding of the disease severity and transmission of the Alpha variant, and to evaluate population-based screening as an intervention to reduce virus transmission.

## Methods

### Population, case definitions, and epidemiological investigation

Lansingerland is a municipality in the region Rotterdam-Rijnmond in the Netherlands, with 63,338 inhabitants (January 1^st^ 2021, www.cbs.nl). School X is located in Lansingerland and has two locations. It shares facilities with two primary schools, five after-school programs, and two day care centres for children under the age of 4 (hereafter ‘neighbouring schools’). Cases were individuals with a SARS-CoV-2 laboratory confirmed by RT-PCR or rapid antigen assay and were residents of Lansingerland (between November 16^th^ 2020 until February 22^th^ 2021), or had an epidemiological link to School X (November 16^th^ 2020 until January 1^st^ 2021), or the neighbouring schools (November 16^th^ 2020 until January 11^th^ 2021). Alpha and non-Alpha variant cases were cases confirmed by additional laboratory testing (variant PCR or whole genome sequencing). Symptomatic or post-exposure testing (routine testing) and population-based screening were performed in four phases based on the course of the outbreak (Table 1). Phase 1 targeted individuals with an epidemiological link to School X and their household members, and phase 2 targeted individuals with an epidemiological link to neighbouring schools of School X and their household members. Phases 3 (voluntary population-based screening) and 4 (post-outbreak monitoring) targeted all residents of Lansingerland. We distinguished cases identified through routine testing (A), and those who were found through voluntary population-based screening (B). Individuals with multiple test results were only included once in the analysis with priority given to the first positive test result. Ethical approval was not required for this study since outbreak investigations of notifiable diseases are a legal task of the Public Health Service as described under the national Public Health Act.

**Table 1:** Number of cases stratified by study phase from November 16^th^ 2020 to February 22^nd^ 2021.

Phase	Period	Definitions	Routine / population-based testing	Approach	Negative	Alpha		non-Alpha		variant unknown		Total cases	Total cases / tested individuals
					**n**	**n**	**%**	**n**	**%**	**n**	**%**	**n**	**%**
1A	Nov 16^th^ 2020 –Jan 1^st^ 2021	Epidemiological link to school X	Routine	Test facility	unknown^±^	61	62,9%	8	8,2%	28	28,9%	97	-
1B	Dec 30^th^ 2020 –Jan 1^st^ 2021	Epidemiological link to school X	Population-based	Screening households	650	16	57,1%	6	21,4%	6	21,4%	28	4,1
2A	Nov 16^th^ 2020 –Jan 11^th^ 2021	Epidemiological link to neighbouring schools*	Routine	Test facility	unknown^±^	12	42,9%	1	3,6%	15	53,6%	28	-
2B	Jan 6^th^ 2021 -Jan 11^th^ 2021	Epidemiological link to neighbouring schools*	Population-based	Screening households	1193	4	25,0%	8	50,0%	4	25,0%	16	1,3
3A	Nov 16^th^ 2020 -Jan 22^nd^ 2021	Living in Lansingerland	Routine	Test facility	2585	34	2,3%	277	18,8%	1160	78,9%	1471	36
3B	Jan 11^th^ 2021 -Jan 22nd 2021	Living in Lansingerland	Population-based	Community screening	36338	13	6,6%	93	47,4%	90	45,9%	196	0,5
4	Jan 23^rd^ 2021 -Feb 22^nd^ 2021	Living in Lansingerland	Routine	Test facility	1063	99	37,4%	57	21,5%	109	41,1%	265	20
			**Total**		**41829**	**239**	**11,4%**	**450**	**21,4%**	**1412**	**67,2%**	**2101**	**4,8**

*Phase 2 neighbouring schools include two primary schools, 5 after-school programs, and 2 child day care centres neighbouring School X

^±^The number of test negative individuals is not known for phase 1A and 2A since contact tracing was only performed for positive cases. Test negative individuals residential in Lansingerland and with an epidemiological link to school X or phase 2 schools were added to phase 3A.

### National guidelines for routine SARS-CoV-2 testing and contact tracing

SARS-CoV-2 testing (RT-PCR or rapid antigen test) was provided free of charge by the regional Public Health Service (PHS) to individuals with COVID-19 like symptoms or after recent contact with a confirmed case. During the study period, national guidelines did not advise testing of children younger than 7 years (due to it being an invasive procedure). Children age 7–12 years were tested when symptomatic (fever, chest tightening), or after recent contact with a confirmed case. As a result, routine testing was biased towards children ≥7 years of age. Contact tracing was performed by phone interview to individuals with lab-confirmation of SARS-CoV-2 infection to assess symptom onset and to make an inventory of contacts.

### Population-based screening and questionnaire

School children, staff, and their household members were invited by email for free of charge SARS-CoV-2 testing (phase 1B and 2B). For the population-based screening of the Lansingerland municipality, all residents received a personal letter and were informed through a town hall meeting chaired by the mayor which was broadcasted on regional and national media (phase 3B). Individuals in phase 1B and 2B were invited by email to fill in a questionnaire including name, address, date of birth, school, classroom, date of symptom onset, and COVID-19 symptoms.

### SARS-CoV-2 diagnostics, S gene target failure, and whole genome sequencing

Nasopharyngeal/throat specimens were obtained by trained personnel at a SARS-CoV-2 test facility and specimens were sent to routine diagnostic laboratories for RT-PCR or tested by trained personnel using a rapid antigen test. Residual positive specimens (collected for either RT-PCR or rapid antigen test) obtained from individuals with an epidemiological link to School X, neighbouring schools, and residents of Lansingerland were requested from the diagnostic laboratories during the study period and tested using the TaqPath RTqPCR (ThermoFisher, Waltham, Massachusetts, United States) for S gene target failures (SGTF). Specimens with N and/or Orf1ab targets below cycle threshold (Ct) value 32, and negative for S were considered as SGTF. SGTF specimens below Ct value 32 were confirmed by whole genome sequencing (WGS) or N501Y PCR to determine the variant. WGS was performed using the Nanopore platform (Oxford Nanopore Technologies, Oxford, UK) or Illumina platform (Illumina, California, US) as described previously [9, 10].

### SARS-CoV-2 detection in sewage

Wastewater specimen were collected at pumping station Bergschenhoek (a village with 18.250 inhabitants within the municipality Lansingerland), and Feijenoord (neighbourhood of the nearby city Rotterdam) as reference. Specimens were collected three days per week as 24h flow-dependent composite samples and processed as previously described [11]. The levels of SARS-CoV-2 were determined and normalized to the proportion of domestic sewage in the samples [12].

### Data and statistical analyses

The regional database with personal identifiers, SARS-CoV-2 test results, symptom onset, and symptoms was merged with the questionnaire and WGS databases. Data were analysed using SPSS Statistics for Windows, version 25 (IBM, Armonk, NY) and R version 4.1.0. Households with at least one individual tested in population-based phases were included in the household analysis (test results of all phases were included for these households). We assumed that all members of a household were infected by the same variant when not all household members’ specimens were available for variant testing. Households with infections with multiple variants were excluded from the analysis. Differences between groups were tested by t-test for continuous variables or chi-square for proportions. Confidence intervals were calculated using the Wilson method. Data obtained in this outbreak investigation could not be shared with a public repository due to the privacy and confidentiality of the individuals involved in this outbreak.

### Sequence analysis

The raw sequence reads were demultiplexed using Porechop (https://github.com/rrwick/Porechop) and processed as previously described for Nanopore [9]. For Illumina sequencing adapter sequences and primer sequences were trimmed, sequence reads were mapped against the GISAID sequence EPI_ISL_412973, and consensus sequences were generated in Geneious v.9.1.8. All Alpha variant sequences from December 12^th^ 2020 until February 22^nd^ 2021 from the Netherlands and sequences from 21 September 2020 until 21 October 2020 from the UK were downloaded from the GISAID database [13]. Identical (or nearly identical) Dutch sequences (maximum 2 nucleotides difference) within the same collection month were removed using an in-house developed R script (https://github.com/dnieuw/alireduce). The remaining sequences were aligned with the outbreak sequences using MUSCLE [14]. The alignment was manually checked for discrepancies, and IQ-TREE was used to perform a maximum-likelihood phylogenetic analysis under the GTR + F + I + G4 model as the best predicted model using the ultrafast bootstrap option with 1,000 replicates [15]. The phylogenetic tree was visualized using FigTree (http://tree.bio.ed.ac.uk/software/figtree/). A cut off of maximal 2 nucleotides was used for sequence cluster detection based on the virus mutation rate and sequence diversity detected in previous outbreaks [16, 17]. The SARS-CoV-2 lineage was determined using the Pangolin tool [18].

## Results

### Outbreak detection

On December 12^th^ 2020, a cluster of SARS-CoV-2 cases was detected at a school with two locations in Lansingerland (School X). The affected school was closed on the same day to prevent further transmission. On December 23^rd^, routine random sequencing of samples as part of the genomic surveillance effort showed the presence of the Alpha virus variant in a patient tested through one of the test facilities operated by the regional PHS. The patient was indirectly linked to School X and this link suggested that the outbreak at School X was caused by the Alpha variant. Analysis of case notifications by postal code showed that the number of positive samples per 100,000 residents in Lansingerland in December 2020 were elevated compared to the rest of the Rotterdam-Rijnmond region (S1 Material). There were no other outbreaks reported to the PHS at the time.

### Outbreak school X

**Fig 1:**
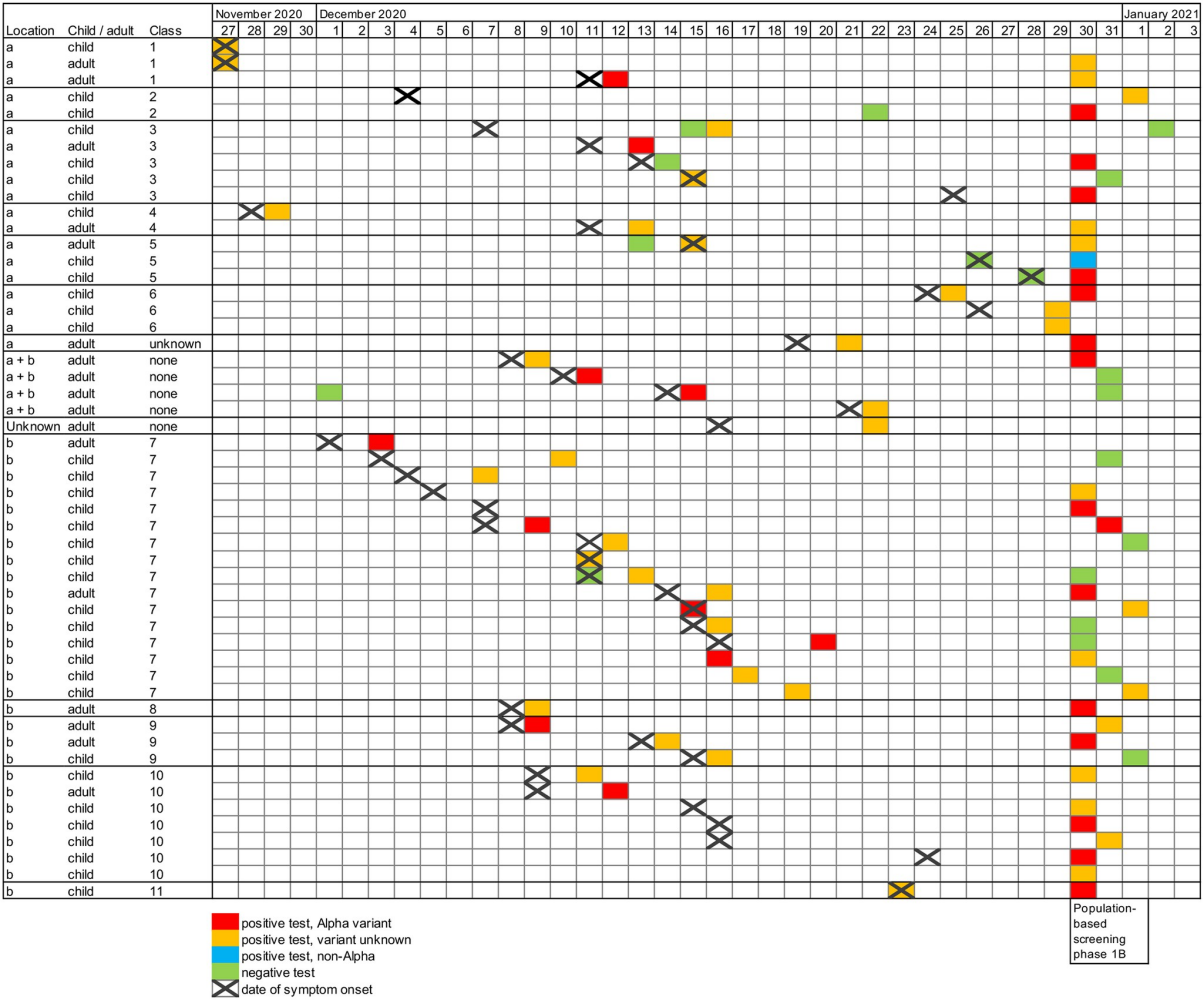
Timeline of cases at School X by classroom and school location in Lansingerland. Each line represents one case with symptom onset (black diagonal cross) and sampling date with variant (coloured box). School children and staff of School X were voluntarily tested regardless of symptoms between December 30^th^ 2020 and January 1^st^ 2021 (phase 1A and 1B).

Routine testing and population-based screening of children and staff of School X, and their household members (phases 1A and 1B) detected a total of 125 SARS-CoV-2 cases: 35 children, 17 staff members at School X and 73 household members (Table 1, Fig 1). Molecular typing showed that 77 out of 91 (85%) SARS-CoV-2 cases with a link to School X for which genotyping could be done were infected with the Alpha variant in phases 1A and 1B.

**Fig 2:**
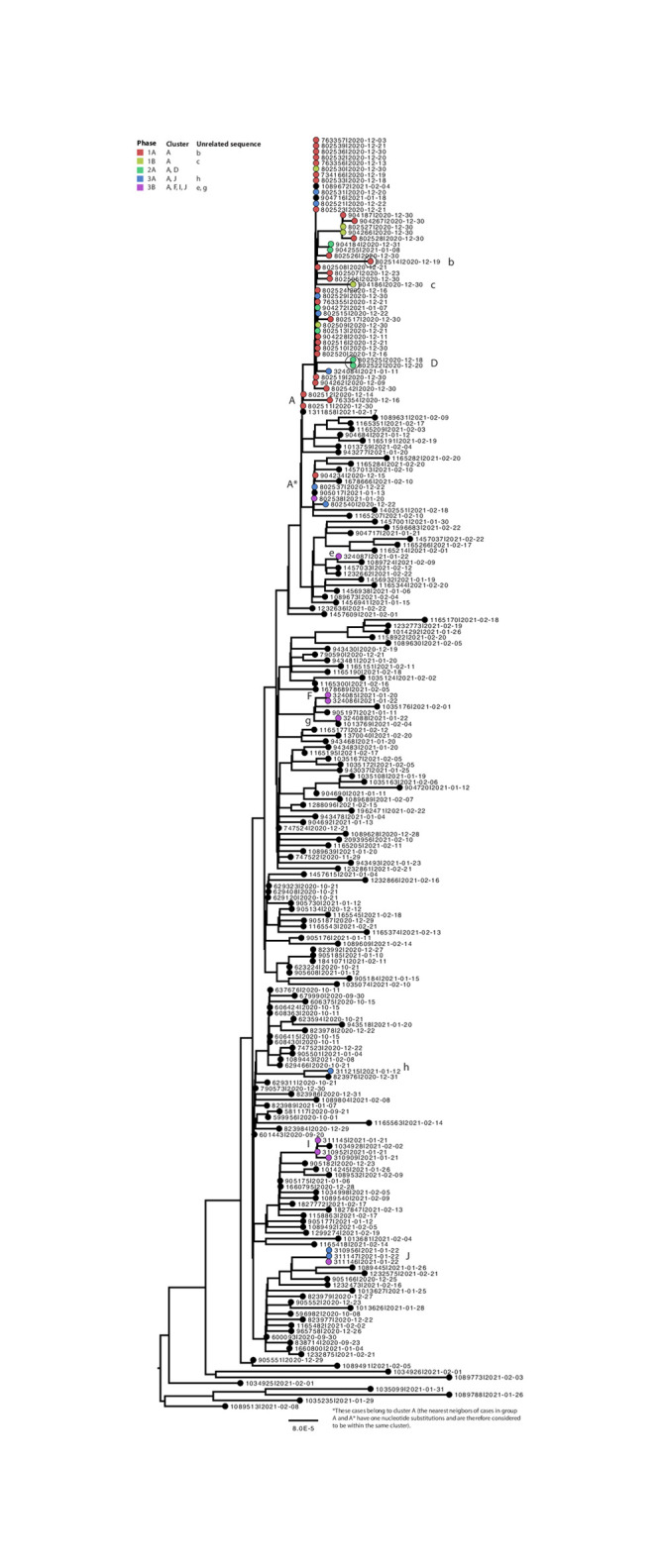
Maximum likelihood analysis of 60 Alpha variant SARS-CoV-2 whole genome sequences obtained from individuals living in the municipality Lansingerland, the Netherlands, or with an epidemiological link to School X or neighbouring schools and child care centres from December 30^th^ 2020 to January 22^nd^ 2021, in a background of 140 Alpha variant sequences obtained from the Netherlands from November 29^th^ 2020 until February 22^th^ 2021 and 20 Alpha variant sequences from the United Kingdom from September 21^th^ until October 21^th^ 2020 (black). Colours indicate the study phase in which specimens were collected. Scale bar represents the number of nucleotide substitutions per site. The text next to each sequence indicates the GISAID accession id and sample collection date.

The symptom onset of the first two cases, a staff member and a child, was November 27^th^ 2021 at School X in classroom 1. The outbreak subsequently spread to a total of 11 classrooms at both locations (a and b) of School X with 1 to 16 cases per classroom (Fig 1). Out of 6 classrooms at location a, a child was the first symptomatic case in 4 classrooms, an adult was the first symptomatic case in 1 classroom, and in 1 classroom both a child and adult were symptomatic first. The first detected symptomatic case at school location b was an adult, which suggests that the staff of School X most likely transmitted the virus from location a to b. Out of 5 classrooms at location b, a child was the first symptomatic case in 1 classroom, an adult was the first symptomatic case in 3 classrooms, and in 1 classroom both a child and adult were symptomatic first. At least eight staff members worked at both locations, of whom four staff members tested positive. Of 18 households with children at both locations, at least four households had one or more positive cases. Of note, 5 symptomatic SARS-CoV-2 cases diagnosed as part of the outbreak in School X initially tested negative by routine testing (4 by rapid antigen test and 1 by RT-PCR). WGS analysis showed that 33 out of 35 (94%) Alpha variant sequences derived from specimens collected in phase 1A and 1B belonged to a cluster A (Fig 2), confirming the outbreak at School X and transmission to household members. The cases with a sequence indicated with b and c (household contact of a case of School X and child at School X, respectively) were considered unrelated.

### Outbreak phase 2 neighbouring schools

After three teachers at a neighbouring primary school tested positive for the Alpha variant on December 31^st^ 2020, retrospective case finding of routinely tested individuals and population-based screening were extended to children and staff of 2 neighbouring primary schools, 5 afterschool programs, and 2 child day care centres neighbouring School X (hereafter ‘neighbouring schools’), and their household members (phase 2A and 2B). A total of 44 SARS-CoV-2 cases were detected with a link to the neighbouring schools. 25 samples were successfully sequenced or typed by variant PCR, of which 16 (64%) were confirmed Alpha variant cases (Table 1), suggesting the Alpha variant spread from School X to the neighbouring schools. These 16 cases infected with Alpha variant were four staff members, four children, and eight household members. WGS analysis showed that four out of six available full genome sequences belonged to the same sequence cluster as School X (cluster A), supporting the hypothesis that cases with an epidemiological link to School X transmitted the virus to cases with a link to the neighbouring schools (Fig 2). Two out of six Alpha variant sequences obtained in phase 2A (cluster D) had three identical mutations when compared to cluster A, potentially reflecting a separate introduction.

### Lansingerland municipality

The rapid spread of the Alpha variant among individuals of both schools and their household members raised the question to which extent the Alpha variant was circulating in the community of Lansingerland. Therefore, samples from SARS-CoV-2 cases confirmed by routine testing were retrieved for variant typing from November 16^th^ 2020 until January 22^nd^ 2021 (phase 3A). The Alpha variant was retrospectively detected in 34 out of 311 (11%) positive specimens for which genotyping could be done and which had no (known) epidemiological link to the School X or neighbouring schools (Table 1). Alpha variant sequences obtained through this approach (n = 10) were more diverse, grouping in clusters A and J, and unrelated sequence h (Fig 2). All remaining residents of Lansingerland were subsequently invited for testing from January 11^th^ until 22^nd^ 2021 (Phase 3B), and 36,534 of 63,338 (58%) residents were tested. 196 out of 36,534 (0,5%) residents tested positive. Thirteen out of 106 (12%) cases for which genotyping could be done were infected by the Alpha variant (Table 1). Out of 9 available Alpha variant Phase B sequences, only 1 sequence clustered with sequences from School X; other sequences were found in clusters F, I, and J, or as unrelated sequences e and g (Fig 2). The sequence results of phases 3A and 3B combined indicate that the Alpha variant had been introduced multiple times in the municipality of Lansingerland until January 22^nd^ 2021.

**Fig 3:**
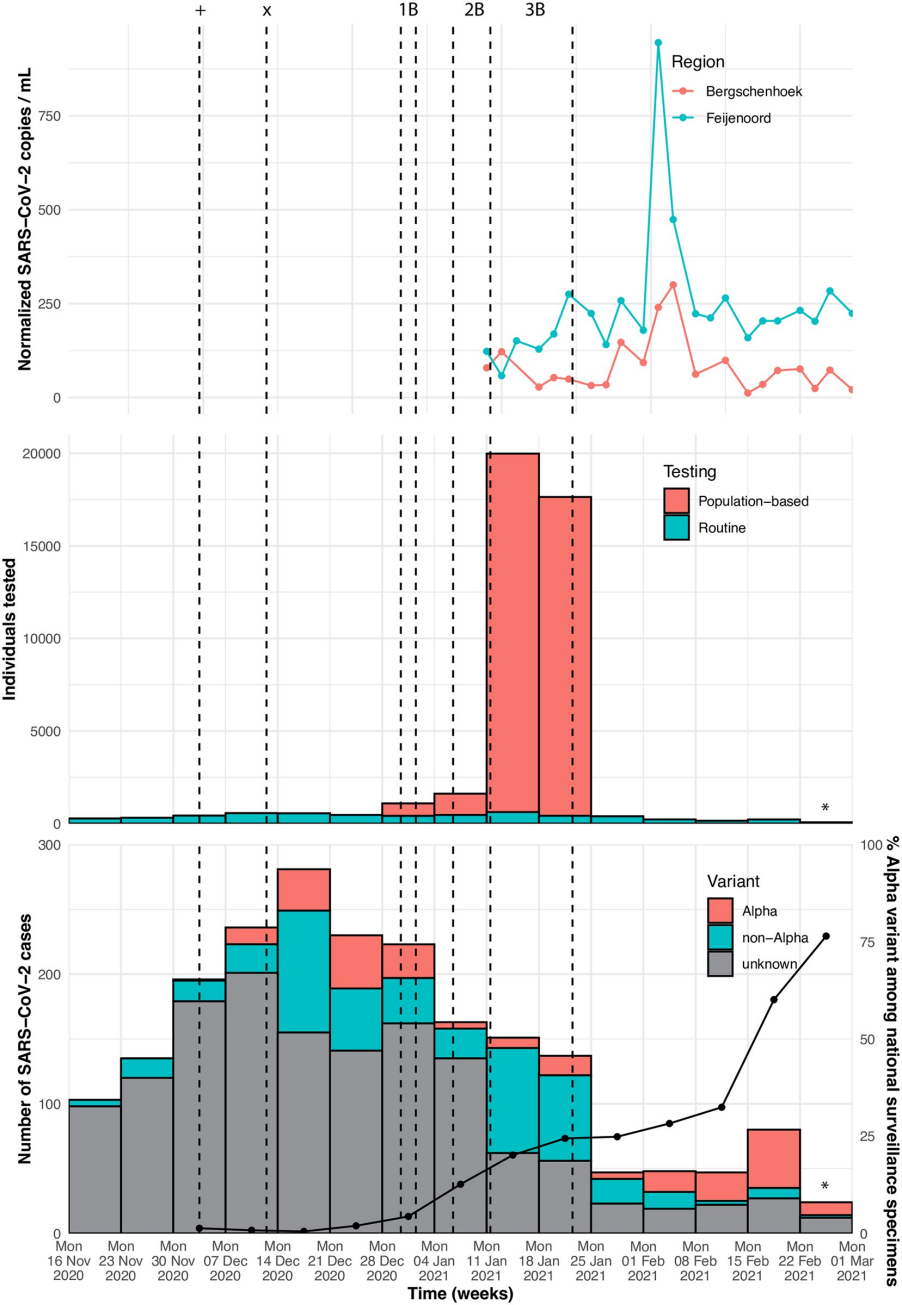
Number of confirmed SARS-CoV-2 cases per week in Lansingerland (including individuals with an epidemiological link to School X or neighbouring schools) by variant (bottom). Line shows the percentage of Alpha variant among national surveillance specimens per week on the right y-axis (data obtained via https://www.rivm.nl/coronavirus-covid-19/virus/varianten) (bottom). Number of individuals tested for SARS-CoV-2 in Lansingerland (including individuals with an epidemiological link to School X or neighbouring schools) (middle). Levels of normalized SARS-CoV-2 copies per mL of domestic wastewater in Bergschenhoek (village in Lansingerland) and Feijenoord (neighbourhood in Rotterdam) (top). Domestic wastewater data were only available from January 11^th^ 2021 onwards. Vertical dashed lines indicate: (+) First confirmed case with Alpha variant at School X, (x) School X closed, (1B) Period of population-based screening at School X (phase 1B), (2B) Period of population-based screening neighbouring schools (phase 2B), (3B) Period of population-based screening residents Lansingerland (phase 3B). *Data incomplete for this week.

After the population-based screening campaign, enhanced surveillance was done to continue monitoring the circulation of the Alpha virus variant. SARS-CoV-2 positive specimens from routinely tested residents of Lansingerland were requested from diagnostic laboratories and tested by variant PCR to monitor the circulation of the Alpha variant in Lansingerland from January 23^rd^ 2021 until February 22^nd^ 2021 (phase 4). Of 265 of these SARS-CoV-2 cases, 176 specimens (66%) could be retrieved for variant testing. The Alpha variant was detected at low levels (5 out of 24 [21%] of cases that had sufficient loads for variant typing) in week 4 (January 25^th^ to 31^st^ 2021), but increased to 45 out of 53 (85%) cases with sufficient loads for genotyping in week 7 (February 15^th^ to 21^st^ 2021) (Fig 3 bottom). Variant replacement in Lansingerland was 1 to 2 weeks ahead compared to national surveillance data (Fig 3 bottom). Positive specimens in phase 4 were tested by variant PCR and were not included in the sequence analysis.

Levels of SARS-CoV-2 in waste water were quantified to study SARS-CoV-2 circulation independent of testing strategy in Lansingerland, and compared to a different community within the city of Rotterdam (Feijenoord) during and after phase 3B. The normalized SARS-CoV-2 RNA copies per mL in Lansingerland followed a similar trend compared to Feijenoord at lower levels, suggesting less virus circulation in Lansingerland compared to Feijenoord from January 11^th^ 2021 onwards (Fig 3 top).

### Clinical symptoms and secondary attack rate

The population-based testing allowed us to make comparisons of the clinical symptoms and secondary attack rate between Alpha and non-Alpha infected individuals. Of 478 cases with available data on symptoms and virus variant, 97 out of 127 (76%) Alpha variant infected cases and 256 of 351 (73%) non-Alpha infected cases reported to be symptomatic (no significant difference, p = 0.449). The mean number of reported symptoms was 2.1 and 2.3 for respectively Alpha and non-Alpha infected symptomatic cases (p = 0.442). Stratification of the analysis by age group did not show significant differences in reported clinical symptoms among age groups (S2 Material).

Households with prospectively tested cases and for whom variant typing could be done were selected to determine the household secondary attack rate (Table 2). The mean age among household members with infection(s) with the Alpha variant was significantly younger compared to household members with non-Alpha variant infection(s) (p = 0.019). There were significantly more children in the households infected with the Alpha variant (p = 0.003) and significantly more adults in non-Alpha infected households (p<0.001). Of 100 individuals at risk in Alpha variant infected households, 41 (41%, 95% confidence interval (CI) 32%-51%) individuals became infected, and 95 of 346 (27%, 95% CI 23%-32%) individuals at risk in non-Alpha variant infected households became infected; a 52% higher transmission among contacts in Alpha variant infected households (p = 0.010).

**Table 2:** Characteristics of households and household members infected by Alpha versus non-Alpha variants.

	Alpha	non-Alpha	P
Households (N)	37	136	
Households with transmission (n)	21	63	
Mean household size	3.7	3.5	0.410
Mean number of children in the household*	1.6	1.0	0.003
Mean number of adults in the household	2.1	2.5	<0.001
Overall household SAR (95% CI)	0.57 (0.41–0.71)	0.46 (0.38–0.55)	0.260
Total household members (N)	137	482	
Phase 1A (n, %)	28 (20)	6 (1.2)	
Phase 1B (n, %)	40 (29)	28 (5.8)	
Phase 2A (n, %)	7 (5.1)	1 (0.2)	
Phase 2B (n, %)	11 (8.0)	18 (3.7)	
Phase 3A (n, %)	14 (10)	148 (31)	
Phase 3B (n, %)	37 (27)	281 (58)	
Mean age household members	29.8	34.2	0.019
Individuals at risk (N)	100	346	
Secondary cases (n)	41	95	
Secondary attack rate (95% CI)	0.41 (0.32–0.51)	0.27 (0.23–0.32)	0.010
Index cases + secondary cases (N)	78	231	
Mean age cases	31.5	34.5	0.239
Information symptoms available (n, %)	67 (86)	204 (88)	0.575
Symptomatic (n/N, %)	48/67 (72)	139/204 (68)	0.590

SAR: secondary attack rate

*<18 years of age

Two households were excluded since both variants were detected. Among 37 Alpha variant infected households, 21 households (57%, 95% CI 41%-71%) showed transmission to at least one contact, which was 24% higher than for non-Alpha variant households (63/136; 46%, 95% CI 38%-55%). This difference was not significant (p = 0.260). The household size was not significantly different between Alpha and non-Alpha variant infected households (p = 0.410).

## Discussion

The outbreak at school X and neighbouring schools resulted in a high number of cases among children and staff, and their household members. Sequencing analysis showed that a single introduction of the Alpha variant drove the outbreak at the school since the majority of sequences obtained from staff members and children of school X belonged to the same sequence cluster. Non alpha variant lineages were detected among the household members of phase 1A and 1B and one child of school X, but these introductions did not result in large-scale transmission and this might be illustrative for a higher transmissibility of the Alpha variant in this setting. Staff members were likely an important driver of virus spread from School X location a to b, and from School X to the neighbouring schools, as the first recognized cases in these locations were adults. At the same time, families had children at both school locations of School X, which may have facilitated transmission between the locations. The Alpha variant school outbreak in Lansingerland showed that large numbers of children become infected at school. At the time of the outbreak, child-to-adult transmission was considered to be less common in school settings than child-to-child transmission [19, 20]. Based on the initial observations from the UK, the contribution of schools in transmission appeared to be different for the Alpha virus variant, although biases from increased testing and outbreak investigations in response to the variant emergence could also explain this observation.

One of the aims of the population-based screening was to assess its utility to track an emerging variant. However, for that specific purpose, retrospective case finding was more successful in finding cases compared to prospective population-based screening: 107 out of 140 (76%) Alpha variant cases were detected using retrospective case finding in phase 1 to 3 (Table 1). Cases infected at School X prior to the closure of the school (December 12^th^ 2020) may have no longer tested positive by the time we did the population-based screening (December 30^th^ 2020 until January 1^st^ 2021). Among population-based screening phases, phase 1B was most effective in finding the highest absolute number of Alpha variant cases and most efficient in terms of the test positive proportion, suggesting that population-based screening is most useful on a targeted population soon after virus introduction. Based on a site specific multi linear regression model that relates SARS-CoV-2 RNA levels in sewage to the number of positive specimens detected by routine testing [21], it has been calculated that the relatively low gene counts in the sewage during phase 3B correspond to 38% of the positive specimens (routinely tested + population-based screening) in Bergschenhoek (village within Lansingerland). This indicates that the population-based community screening revealed an additional 62% of positive cases which would otherwise not have been detected.

From the combined data, we conclude that the population-wide screening and communication efforts had a temporary effect on lowering the number of infectious individuals in the municipality. Sequence analysis showed that the outbreak strain was only detected in the community (individuals without a known link to the schools) in phase 3A, and only detected once in phase 3B, suggesting that transmission of the outbreak strain was almost fully interrupted prior to the population-based screening of the municipality. The population-based screening phase 1 may have helped to stop the outbreak, together with other national and regional public health measures (national lockdown, closure of schools, general hygiene and quarantine rules, and weekly press conferences with an update on the Alpha variant in Lansingerland). It was shown earlier in Slovakia that the combination of nationwide restrictions and population-based screening with quarantine measures rapidly reduced the case incidence [22]. However, sequence analysis also showed that different strains of the Alpha variant in the Lansingerland municipality were circulating in phase 3B, showing that multiple introductions of the Alpha variant had taken place before the municipality screening effort, possibly due to increased traveling and visiting related to the Christmas holiday or due to intensive trade of horticulture products produced in the Lansingerland municipality with the UK and surrounding countries. Therefore, the emergence of the Alpha variant in the municipality was merely delayed by the population-based screening.

We detected five cases with a sequence with three nucleotide substitutions compared to the outbreak cluster A (sequences b, c, e, and both sequences in cluster D, Fig 2) and we concluded that these cases are likely unrelated to the outbreak based on the cluster definition of a maximum of 2 nucleotide substitutions. However, we cannot completely rule out that the genetic gap between cluster A and these sequences were caused by limited sampling coverage (sequences b, c, and both sequences in cluster D), or limited sampling coverage and time between sample collection (sequence e).

No significant differences were observed in the number of reported symptoms between Alpha variant and non-Alpha variant infected individuals in this study. One study reported increased disease severity, risk of hospitalization, or case fatality among Alpha variant cases [23], while another one did not find a relationship between the Alpha variant and disease severity [24]. In our study, most cases showed mild symptoms, and the Alpha variant population was skewed towards younger age groups due to the primary school setting of the outbreak. As COVID-19 symptoms in children were typically mild, the national guidelines did advise to limit testing of primary school children. This may have led to an underestimation of the cases in the younger age groups of School X in the retrospective screening phases (all age groups got the opportunity for a test in the prospective screening phases). We may also have slightly underestimated the number of participants in the population-based screening phases since we cannot exclude that some individuals who wanted to participate in the screening have made an appointment at a routine test facility rather than the designated test street locations for population-based screening.

We showed that the secondary attack rate (SAR) of Alpha variant infected households was higher compared to non-Alpha households and this supports the higher transmissibility of the Alpha variant as found in other studies [25, 26]. However, the SAR obtained in this study needs to be interpreted with caution since we found the mean household age, and mean number of children and adults per household as confounding factors. We can also not rule out that the higher SAR for the Alpha variant in this study is due to timing of the cross-sectional testing of the households. The majority of the Alpha variant infected households were tested a few weeks after the outbreak spread at School X, while the exposure date was unknown for non-Alpha variant infected households.

Understanding essential traits of emerging SARS-CoV-2 variants is a critical component of the global public health response, and epidemiological studies are recommended to provide estimates of SAR, clinical severity, and the contribution of different age groups in transmission. We describe a large-scale effort aimed at collecting such evidence. However, to be useful to inform risk assessment, the ability to collect such data in real-time needs to be built into future public health preparedness through an embedded outbreak research agenda.

## Supporting information

S1 Material Number of positive SARS-CoV-2 samples per 100,000 residents per week in Lansingerland and the rest of the Rotterdam-Rijnmond region from July 1^st^ 2020 through February 22^nd^ 2021. (DOCX)

S2 Material Stratification of symptoms per age group and variant. (DOCX)

S3 Material Acknowledgement viral sequences obtained by external laboratories. (PDF)
