# Supplementary material for: Population-based screening in a municipality after a primary school outbreak of the SARS-CoV-2 Alpha variant, the Netherlands, December 2020–February 2021
Source: PLoS One. 2022 Oct 27;17(10):e0276696. doi: 10.1371/journal.pone.0276696 (PMC9612486; doi:10.1371/journal.pone.0276696)
Supplement: S1 Material — (DOCX) [file pone.0276696.s001.docx]

**S1 Material** Number of positive SARS-CoV-2 samples per 100,000 residents per week in Lansingerland and the rest of the Rotterdam-Rijnmond region from July 1^st^ 2020 through February 22^nd^ 2021. Week 53 is extrapolated to 7 days. First confirmed Alpha variant case, timing of school closure, and start of testing phases is indicated by vertical dashed lines. a: First confirmed case with Alpha variant at School X, b: School A closed, c: Testing School A begins, d: Testing surrounding schools begins, e: Testing entire municipality begins, f: Testing entire municipality ends.
