# Supplementary material for: Population-based screening in a municipality after a primary school outbreak of the SARS-CoV-2 Alpha variant, the Netherlands, December 2020–February 2021
Source: PLoS One. 2022 Oct 27;17(10):e0276696. doi: 10.1371/journal.pone.0276696 (PMC9612486; doi:10.1371/journal.pone.0276696)
Supplement: S2 Material — (DOCX) [file pone.0276696.s002.docx]

**S2 Material** Stratification of symptoms per age group and variant

|  | 0-12 | |  | 13-18 | |  | 19+ | |
| --- | --- | --- | --- | --- | --- | --- | --- | --- |
|  | Alpha  (n=29) | Non-Alpha  (n=31) |  | Alpha  (n=7) | Non-Alpha  (n=46) |  | Alpha  (n=70) | Non-Alpha  (n=235) |
| Symptomatic | 48% | 26% |  | 43% | 41% |  | 76% | 49% |
| Asymptomatic | 41% | 45% |  | 29% | 41% |  | 19% | 37% |
| Unknown | 10% | 29% |  | 29% | 17% |  | 6% | 13% |
|  |  |  |  |  |  |  |  |  |
| Of those with symptoms |  |  |  |  |  |  |  |  |
| Runny/stuffy nose | 36% | 100% |  | 33% | 58% |  | 42% | 55% |
| Cough | 14% | 25% |  | 0% | 53% |  | 64% | 53% |
| Loss of smell | 14% | 25% |  | 0% | 16% |  | 15% | 7% |
| Loss of taste | 0% | 0% |  | 0% | 16% |  | 13% | 9% |
| Sore throat | 14% | 25% |  | 67% | 53% |  | 36% | 36% |
| Chest tightness | 0% | 0% |  | 0% | 5% |  | 15% | 11% |
| Fever | 29% | 50% |  | 0% | 32% |  | 38% | 22% |
| Something else | 36% | 38% |  | 33% | 16% |  | 42% | 22% |
