# Supplementary material for: Population-based screening in a municipality after a primary school outbreak of the SARS-CoV-2 Alpha variant, the Netherlands, December 2020–February 2021
Source: PLoS One. 2022 Oct 27;17(10):e0276696. doi: 10.1371/journal.pone.0276696 (PMC9612486; doi:10.1371/journal.pone.0276696)
Supplement: S3 Material — (PDF) [file pone.0276696.s003.pdf]

We gratefully acknowledge the following Authors from the Originating laboratories responsible for obtaining the specimens, as well as the Submitting laboratories where the genome data were generated and shared via GISAID, on which this research is based.

All Submitters of data may be contacted directly via www.gisaid.org

Authors are sorted alphabetically.

Accession ID	Originating Laboratory	Submitting Laboratory	Authors
EPI_ISL_1678591, EPI_ISL_1678593, EPI_ISL_1678606, EPI_ISL_1678608, EPI_ISL_1678614, EPI_ISL_1678615, EPI_ISL_1678616, EPI_ISL_1678626, EPI_ISL_1678629, EPI_ISL_1678631, EPI_ISL_1678633, EPI_ISL_1678634, EPI_ISL_1678636, EPI_ISL_1678638, EPI_ISL_1678642, EPI_ISL_1678644, EPI_ISL_1678645, EPI_ISL_1678646, EPI_ISL_1678647, EPI_ISL_1678649, EPI_ISL_1678651, EPI_ISL_1678657, EPI_ISL_1678658, EPI_ISL_1678661, EPI_ISL_1678662, EPI_ISL_1678663, EPI_ISL_1678666, EPI_ISL_1678669, EPI_ISL_1678671, EPI_ISL_1678676, EPI_ISL_1678677, EPI_ISL_1678681, EPI_ISL_1678682, EPI_ISL_1678686, EPI_ISL_1678687, EPI_ISL_1678688, EPI_ISL_1678689, EPI_ISL_1678693, EPI_ISL_1678694, EPI_ISL_1678697, EPI_ISL_1678699, EPI_ISL_1678701, EPI_ISL_1678702, EPI_ISL_1678703, EPI_ISL_1678705, EPI_ISL_1678709, EPI_ISL_1678710, EPI_ISL_1678712, EPI_ISL_1678713, EPI_ISL_1678715, EPI_ISL_1678716, EPI_ISL_1678735			
see above	Department of Medical Microbiology & Infection prevention, Amsterdam University Medical Centers location AMC	Department of Medical Microbiology & Infection prevention, Amsterdam University Medical Centers location AMC	Fokla Zorgdrager; Janke Schinkel; Marcel Jonges; Matthijs Welkers; Menno de Jong; Robin van Houdt; Sebastien Matamoros; Sjoerd Rebers
EPI_ISL_734166, EPI_ISL_763304, EPI_ISL_763354, EPI_ISL_763355, EPI_ISL_763356, EPI_ISL_763357, EPI_ISL_801422, EPI_ISL_802506, EPI_ISL_802507, EPI_ISL_802508, EPI_ISL_802509, EPI_ISL_802510, EPI_ISL_802511, EPI_ISL_802512, EPI_ISL_802513, EPI_ISL_802514, EPI_ISL_802516, EPI_ISL_802517, EPI_ISL_802518, EPI_ISL_802519, EPI_ISL_802520, EPI_ISL_802521, EPI_ISL_802522, EPI_ISL_802523, EPI_ISL_802524, EPI_ISL_802525, EPI_ISL_802526, EPI_ISL_802527, EPI_ISL_802528, EPI_ISL_802529, EPI_ISL_802530, EPI_ISL_802531, EPI_ISL_802532, EPI_ISL_802533, EPI_ISL_802536, EPI_ISL_802537, EPI_ISL_802538, EPI_ISL_802539, EPI_ISL_802540, EPI_ISL_802541, EPI_ISL_802542, EPI_ISL_904143, EPI_ISL_904160, EPI_ISL_904184, EPI_ISL_904186, EPI_ISL_904187, EPI_ISL_904196, EPI_ISL_904197, EPI_ISL_904198, EPI_ISL_904199, EPI_ISL_904200, EPI_ISL_904201, EPI_ISL_904202, EPI_ISL_904203, EPI_ISL_904204, EPI_ISL_904205, EPI_ISL_904206, EPI_ISL_904207, EPI_ISL_904208, EPI_ISL_904209, EPI_ISL_904210, EPI_ISL_904211, EPI_ISL_904212, EPI_ISL_904213, EPI_ISL_904214, EPI_ISL_904215, EPI_ISL_904216, EPI_ISL_904217, EPI_ISL_904218, EPI_ISL_904219, EPI_ISL_904220, EPI_ISL_904231, EPI_ISL_904232, EPI_ISL_904234, EPI_ISL_904247, EPI_ISL_904254, EPI_ISL_904255, EPI_ISL_904262, EPI_ISL_904263, EPI_ISL_904264, EPI_ISL_904265, EPI_ISL_904266, EPI_ISL_904267, EPI_ISL_904268, EPI_ISL_904269, EPI_ISL_904270, EPI_ISL_904271, EPI_ISL_904272, EPI_ISL_904273, EPI_ISL_904274, EPI_ISL_904275, EPI_ISL_904276, EPI_ISL_904277, EPI_ISL_904318, EPI_ISL_1310846, EPI_ISL_1310868, EPI_ISL_1310879, EPI_ISL_1310887, EPI_ISL_1310892, EPI_ISL_1310909, EPI_ISL_1310911, EPI_ISL_1310916, EPI_ISL_1310920, EPI_ISL_1310921, EPI_ISL_1310924, EPI_ISL_1310925, EPI_ISL_1310930, EPI_ISL_1310946, EPI_ISL_1310948, EPI_ISL_1310951, EPI_ISL_1310952, EPI_ISL_1310953, EPI_ISL_1310956, EPI_ISL_1310969, EPI_ISL_1310975, EPI_ISL_1310985, EPI_ISL_1310999, EPI_ISL_1311002, EPI_ISL_1311019, EPI_ISL_1311020, EPI_ISL_1311035, EPI_ISL_1311039, EPI_ISL_1311040, EPI_ISL_1311041, EPI_ISL_1311049, EPI_ISL_1311062, EPI_ISL_1311070, EPI_ISL_1311072, EPI_ISL_1311074, EPI_ISL_1311076, EPI_ISL_1311077, EPI_ISL_1311078, EPI_ISL_1311079, EPI_ISL_1311080, EPI_ISL_1311094, EPI_ISL_1311095, EPI_ISL_1311096, EPI_ISL_1311097, EPI_ISL_1311098, EPI_ISL_1311099, EPI_ISL_1311100, EPI_ISL_1311101, EPI_ISL_1311102, EPI_ISL_1311103, EPI_ISL_1311104, EPI_ISL_1311105, EPI_ISL_1311106, EPI_ISL_1311107, EPI_ISL_1311108, EPI_ISL_1311109, EPI_ISL_1311110, EPI_ISL_1311111, EPI_ISL_1311112, EPI_ISL_1311113, EPI_ISL_1311114, EPI_ISL_1311115, EPI_ISL_1311117, EPI_ISL_1311118, EPI_ISL_1311119, EPI_ISL_1311122, EPI_ISL_1311123, EPI_ISL_1311124, EPI_ISL_1311125, EPI_ISL_1311126, EPI_ISL_1311127, EPI_ISL_1311128, EPI_ISL_1311129, EPI_ISL_1311130, EPI_ISL_1311131, EPI_ISL_1311132, EPI_ISL_1311133, EPI_ISL_1311134, EPI_ISL_1311135, EPI_ISL_1311136, EPI_ISL_1311137, EPI_ISL_1311138, EPI_ISL_1311139, EPI_ISL_1311140, EPI_ISL_1311141, EPI_ISL_1311142, EPI_ISL_1311143, EPI_ISL_1311145, EPI_ISL_1311146, EPI_ISL_1311147, EPI_ISL_1311148, EPI_ISL_1311149, EPI_ISL_1311150, EPI_ISL_1311151, EPI_ISL_1311152, EPI_ISL_1311153, EPI_ISL_1311155, EPI_ISL_1311156, EPI_ISL_1311157, EPI_ISL_1311158, EPI_ISL_1311159, EPI_ISL_1311160, EPI_ISL_1311161, EPI_ISL_1311162, EPI_ISL_1311163, EPI_ISL_1311164, EPI_ISL_1311165, EPI_ISL_1311166, EPI_ISL_1311167, EPI_ISL_1311168, EPI_ISL_1311169, EPI_ISL_1311174, EPI_ISL_1311175, EPI_ISL_1311176, EPI_ISL_1311177, EPI_ISL_1311179, EPI_ISL_1311180, EPI_ISL_1311181, EPI_ISL_1311182, EPI_ISL_1311183, EPI_ISL_1311184, EPI_ISL_1311186, EPI_ISL_1311187, EPI_ISL_1311188, EPI_ISL_1311189, EPI_ISL_1311190, EPI_ISL_1311191, EPI_ISL_1311192, EPI_ISL_1311193, EPI_ISL_1311195, EPI_ISL_1311197, EPI_ISL_1311211, EPI_ISL_1311212, EPI_ISL_1311215, EPI_ISL_1311216, EPI_ISL_1311225, EPI_ISL_1311229, EPI_ISL_1311233, EPI_ISL_1311250, EPI_ISL_1311253, EPI_ISL_1311274, EPI_ISL_1311289, EPI_ISL_1311290, EPI_ISL_1311295, EPI_ISL_1311302, EPI_ISL_1311305, EPI_ISL_1324029, EPI_ISL_1324030, EPI_ISL_1324031, EPI_ISL_1324032, EPI_ISL_1324033, EPI_ISL_1324034, EPI_ISL_1324035, EPI_ISL_1324036, EPI_ISL_1324037, EPI_ISL_1324038, EPI_ISL_1324039, EPI_ISL_1324040, EPI_ISL_1324041, EPI_ISL_1324042, EPI_ISL_1324043, EPI_ISL_1324044, EPI_ISL_1324045, EPI_ISL_1324046, EPI_ISL_1324047, EPI_ISL_1324048, EPI_ISL_1324049, EPI_ISL_1324050, EPI_ISL_1324051, EPI_ISL_1324052, EPI_ISL_1324053, EPI_ISL_1324054, EPI_ISL_1324055, EPI_ISL_1324056, EPI_ISL_1324057, EPI_ISL_1324058, EPI_ISL_1324059, EPI_ISL_1324060, EPI_ISL_1324061, EPI_ISL_1324062, EPI_ISL_1324063, EPI_ISL_1324064, EPI_ISL_1324065, EPI_ISL_1324066, EPI_ISL_1324067, EPI_ISL_1324068, EPI_ISL_1324069, EPI_ISL_1324070, EPI_ISL_1324071, EPI_ISL_1324072, EPI_ISL_1324073, EPI_ISL_1324074, EPI_ISL_1324075, EPI_ISL_1324076, EPI_ISL_1324078, EPI_ISL_1324079, EPI_ISL_1324080, EPI_ISL_1324081, EPI_ISL_1324082, EPI_ISL_1324083, EPI_ISL_1324084, EPI_ISL_1324085, EPI_ISL_1324086, EPI_ISL_1324087, EPI_ISL_1324088, EPI_ISL_1324089, EPI_ISL_1324090, EPI_ISL_1324091, EPI_ISL_1324092, EPI_ISL_1324093, EPI_ISL_1324094, EPI_ISL_1324095, EPI_ISL_1324096, EPI_ISL_1324097, EPI_ISL_1324098, EPI_ISL_1324103, EPI_ISL_1324104, EPI_ISL_1324105, EPI_ISL_1324106, EPI_ISL_2145099, EPI_ISL_2145228, EPI_ISL_2145232, EPI_ISL_2145234, EPI_ISL_2145258, EPI_ISL_2145350, EPI_ISL_2145351, EPI_ISL_2145353, EPI_ISL_2145354, EPI_ISL_2145356, EPI_ISL_2145402, EPI_ISL_2145419, EPI_ISL_2145421, EPI_ISL_2145423, EPI_ISL_2145506, EPI_ISL_2145509, EPI_ISL_2145511, EPI_ISL_2145513, EPI_ISL_2145514, EPI_ISL_2145516, EPI_ISL_2145517, EPI_ISL_2145591, EPI_ISL_2145593, EPI_ISL_2145594, EPI_ISL_2145596, EPI_ISL_2145597, EPI_ISL_2145684, EPI_ISL_2145720, EPI_ISL_2145721, EPI_ISL_2145793, EPI_ISL_2145794, EPI_ISL_2154094, EPI_ISL_2154095, EPI_ISL_2154103, EPI_ISL_2154106, EPI_ISL_2154116, EPI_ISL_2154123, EPI_ISL_2154131, EPI_ISL_2154147, EPI_ISL_2154148, EPI_ISL_2154152, EPI_ISL_2154155, EPI_ISL_2154180, EPI_ISL_2154188, EPI_ISL_2154191, EPI_ISL_2154194, EPI_ISL_2154204, EPI_ISL_2154218, EPI_ISL_2154219, EPI_ISL_2154221, EPI_ISL_2154283, EPI_ISL_2154284, EPI_ISL_2154285, EPI_ISL_2154286, EPI_ISL_2154297, EPI_ISL_2154298, EPI_ISL_2154299, EPI_ISL_2154300, EPI_ISL_2154301, EPI_ISL_2154302, EPI_ISL_2154304, EPI_ISL_2154308, EPI_ISL_2154309, EPI_ISL_2154310, EPI_ISL_2154327, EPI_ISL_2154328, EPI_ISL_2154334, EPI_ISL_2154335, EPI_ISL_2154346, EPI_ISL_2154347, EPI_ISL_2154348, EPI_ISL_2154349, EPI_ISL_2154350, EPI_ISL_2449951, EPI_ISL_2449953, EPI_ISL_2449991, EPI_ISL_2449992, EPI_ISL_2483477, EPI_ISL_2483478, EPI_ISL_2483479, EPI_ISL_2483480, EPI_ISL_2483481, EPI_ISL_2483482, EPI_ISL_2483552, EPI_ISL_2483553, EPI_ISL_2483554, EPI_ISL_2483555, EPI_ISL_2483556, EPI_ISL_2483558, EPI_ISL_2483559, EPI_ISL_2860453, EPI_ISL_2860454, EPI_ISL_2860455, EPI_ISL_2860498, EPI_ISL_2860530, EPI_ISL_2860531, EPI_ISL_2860557, EPI_ISL_2887806, EPI_ISL_2887826, EPI_ISL_2887837, EPI_ISL_2887838, EPI_ISL_3135938			
see above	Dutch COVID-19 response team	Erasmus Medical Center	Anne van der Linden; Annemiek van der Eijk; Bas Oude Munnink; Corine GeurtsvanKessel; David Nieuwenhuijse; Emmanuelle Munger; Irina Chestakova; Marion Koopmans; Marjan Boter; Reina Sikkema; Richard Molenkamp; on behalf of the Dutch national COVID-19 respo; on behalf of the Dutch national COVID-19 response team.
EPI_ISL_965559, EPI_ISL_965747, EPI_ISL_965755, EPI_ISL_965758, EPI_ISL_1120186, EPI_ISL_1120228, EPI_ISL_1120277, EPI_ISL_1120310, EPI_ISL_1120372, EPI_ISL_1120386, EPI_ISL_1120415, EPI_ISL_1120469, EPI_ISL_1120490, EPI_ISL_1120491, EPI_ISL_1120517, EPI_ISL_1158591, EPI_ISL_1158597, EPI_ISL_1158602, EPI_ISL_1158604, EPI_ISL_1158605, EPI_ISL_1158606, EPI_ISL_1158614, EPI_ISL_1158615, EPI_ISL_1158623, EPI_ISL_1158635, EPI_ISL_1158662, EPI_ISL_1158663, EPI_ISL_1158669, EPI_ISL_1158673, EPI_ISL_1158677, EPI_ISL_1158680, EPI_ISL_1158684, EPI_ISL_1158686, EPI_ISL_1158690, EPI_ISL_1158703, EPI_ISL_1158726, EPI_ISL_1158727, EPI_ISL_1158735, EPI_ISL_1158739, EPI_ISL_1158741, EPI_ISL_1158760, EPI_ISL_1158766, EPI_ISL_1158776, EPI_ISL_1158785, EPI_ISL_1158795, EPI_ISL_1158796, EPI_ISL_1158803, EPI_ISL_1158804, EPI_ISL_1158805, EPI_ISL_1158806, EPI_ISL_1158808, EPI_ISL_1158819, EPI_ISL_1158821, EPI_ISL_1158826, EPI_ISL_1158835, EPI_ISL_1158838, EPI_ISL_1158841, EPI_ISL_1158848, EPI_ISL_1158852, EPI_ISL_1158853, EPI_ISL_1158856, EPI_ISL_1158859, EPI_ISL_1158861, EPI_ISL_1158863, EPI_ISL_1158864, EPI_ISL_1158865, EPI_ISL_1158867, EPI_ISL_1158869, EPI_ISL_1158872, EPI_ISL_1158875, EPI_ISL_1158876, EPI_ISL_1158878, EPI_ISL_1158880, EPI_ISL_1158881, EPI_ISL_1158882, EPI_ISL_1158889, EPI_ISL_1158890, EPI_ISL_1158891, EPI_ISL_1158892, EPI_ISL_1158893, EPI_ISL_1158895, EPI_ISL_1158896, EPI_ISL_1158897, EPI_ISL_1158900, EPI_ISL_1158901, EPI_ISL_1158922, EPI_ISL_1210354, EPI_ISL_1210355, EPI_ISL_1210356, EPI_ISL_1210357, EPI_ISL_1210361, EPI_ISL_1210365, EPI_ISL_1210366, EPI_ISL_1210367, EPI_ISL_1337765, EPI_ISL_1337795, EPI_ISL_1509643, EPI_ISL_1509649, EPI_ISL_1509651, EPI_ISL_1509653, EPI_ISL_1509697, EPI_ISL_1509698, EPI_ISL_1509699, EPI_ISL_1509700, EPI_ISL_1509701, EPI_ISL_1509717, EPI_ISL_1558594, EPI_ISL_3474258, EPI_ISL_3474303, EPI_ISL_3474317, EPI_ISL_3474337, EPI_ISL_3474380, EPI_ISL_3474456, EPI_ISL_3474460, EPI_ISL_3474464, EPI_ISL_3474467, EPI_ISL_3474478, EPI_ISL_3474482, EPI_ISL_3474488, EPI_ISL_3474501, EPI_ISL_3474503, EPI_ISL_3474505, EPI_ISL_3474515, EPI_ISL_3474593, EPI_ISL_3474594, EPI_ISL_3474598, EPI_ISL_3474602, EPI_ISL_3474625, EPI_ISL_3474631, EPI_ISL_3474715, EPI_ISL_3474716			
see above	Dutch COVID-19 response team	Medical Microbiology, Maastricht University Medical Centre	Brian van der Veer*; Carmen Reumkens; Christian Hoebe; Erik Beuken; Jozef Dingemans*; Lieke van Alphen; Paul Savelkoul
EPI_ISL_728566, EPI_ISL_728568, EPI_ISL_747521, EPI_ISL_747523, EPI_ISL_747524, EPI_ISL_747525, EPI_ISL_790571, EPI_ISL_790572, EPI_ISL_790573, EPI_ISL_790574, EPI_ISL_790580, EPI_ISL_790581, EPI_ISL_790582, EPI_ISL_790583, EPI_ISL_790584, EPI_ISL_790585, EPI_ISL_790586, EPI_ISL_790587, EPI_ISL_790588, EPI_ISL_790589, EPI_ISL_790590, EPI_ISL_790591, EPI_ISL_823976, EPI_ISL_823977, EPI_ISL_823978, EPI_ISL_823979, EPI_ISL_823980, EPI_ISL_823981, EPI_ISL_823982, EPI_ISL_823983, EPI_ISL_823984, EPI_ISL_823985, EPI_ISL_823986, EPI_ISL_823987, EPI_ISL_823988, EPI_ISL_823989, EPI_ISL_823992, EPI_ISL_826523, EPI_ISL_826524, EPI_ISL_826525, EPI_ISL_826526, EPI_ISL_826527, EPI_ISL_826528, EPI_ISL_826529, EPI_ISL_826530, EPI_ISL_826531, EPI_ISL_826532, EPI_ISL_826533, EPI_ISL_826534, EPI_ISL_826535, EPI_ISL_826536, EPI_ISL_904659, EPI_ISL_904660, EPI_ISL_904661, EPI_ISL_904662, EPI_ISL_904663, EPI_ISL_904664, EPI_ISL_904665, EPI_ISL_904666, EPI_ISL_904667, EPI_ISL_904668, EPI_ISL_904669, EPI_ISL_904670, EPI_ISL_904671, EPI_ISL_904674, EPI_ISL_904675, EPI_ISL_904676, EPI_ISL_904677, EPI_ISL_904678, EPI_ISL_904679, EPI_ISL_904680, EPI_ISL_904681, EPI_ISL_904682, EPI_ISL_904683, EPI_ISL_904684, EPI_ISL_904685, EPI_ISL_904686, EPI_ISL_904687, EPI_ISL_904688, EPI_ISL_904689, EPI_ISL_904690, EPI_ISL_904691, EPI_ISL_904692, EPI_ISL_904693, EPI_ISL_904694, EPI_ISL_904695, EPI_ISL_904696, EPI_ISL_904697, EPI_ISL_904698, EPI_ISL_904699, EPI_ISL_904700, EPI_ISL_904701, EPI_ISL_904702, EPI_ISL_904703, EPI_ISL_904704, EPI_ISL_904705, EPI_ISL_904706, EPI_ISL_904707, EPI_ISL_904708, EPI_ISL_904709, EPI_ISL_904710, EPI_ISL_904711, EPI_ISL_904712, EPI_ISL_904713, EPI_ISL_904714, EPI_ISL_904715, EPI_ISL_904716, EPI_ISL_904717, EPI_ISL_904718, EPI_ISL_904719, EPI_ISL_904759, EPI_ISL_904958, EPI_ISL_904959, EPI_ISL_904960, EPI_ISL_904962, EPI_ISL_904963, EPI_ISL_904964, EPI_ISL_904965, EPI_ISL_904966, EPI_ISL_904967, EPI_ISL_904968, EPI_ISL_904969, EPI_ISL_904970, EPI_ISL_904971, EPI_ISL_904976, EPI_ISL_904978, EPI_ISL_904979, EPI_ISL_904980, EPI_ISL_905005, EPI_ISL_905017, EPI_ISL_905022, EPI_ISL_905032, EPI_ISL_905064, EPI_ISL_905068, EPI_ISL_905101, EPI_ISL_905104, EPI_ISL_905114, EPI_ISL_905118, EPI_ISL_905119, EPI_ISL_905122, EPI_ISL_905128, EPI_ISL_905134, EPI_ISL_905138, EPI_ISL_905158, EPI_ISL_905166, EPI_ISL_905169, EPI_ISL_905175, EPI_ISL_905176, EPI_ISL_905177, EPI_ISL_905178, EPI_ISL_905179, EPI_ISL_905180, EPI_ISL_905181, EPI_ISL_905182, EPI_ISL_905183, EPI_ISL_905184, EPI_ISL_905185, EPI_ISL_905186, EPI_ISL_905187, EPI_ISL_905190, EPI_ISL_905191, EPI_ISL_905192, EPI_ISL_905193, EPI_ISL_905194, EPI_ISL_905195, EPI_ISL_905196, EPI_ISL_905197, EPI_ISL_905198, EPI_ISL_905199, EPI_ISL_905200, EPI_ISL_905201, EPI_ISL_905367, EPI_ISL_905376, EPI_ISL_905388, EPI_ISL_905397, EPI_ISL_905401, EPI_ISL_905405, EPI_ISL_905407, EPI_ISL_905417, EPI_ISL_905418, EPI_ISL_905419, EPI_ISL_905424, EPI_ISL_905441, EPI_ISL_905450, EPI_ISL_905451, EPI_ISL_905456, EPI_ISL_905485, EPI_ISL_905488, EPI_ISL_905492, EPI_ISL_905494, EPI_ISL_905501, EPI_ISL_905510, EPI_ISL_905513, EPI_ISL_905515, EPI_ISL_905529, EPI_ISL_905536, EPI_ISL_905537, EPI_ISL_905548, EPI_ISL_905549, EPI_ISL_905550, EPI_ISL_905551, EPI_ISL_905552, EPI_ISL_905556, EPI_ISL_905557, EPI_ISL_905558, EPI_ISL_905559, EPI_ISL_905560, EPI_ISL_905585, EPI_ISL_905590, EPI_ISL_905591, EPI_ISL_905608, EPI_ISL_905614, EPI_ISL_905615, EPI_ISL_905619, EPI_ISL_905621, EPI_ISL_905625, EPI_ISL_905630, EPI_ISL_905661, EPI_ISL_905676, EPI_ISL_905713, EPI_ISL_905718, EPI_ISL_905719, EPI_ISL_905721, EPI_ISL_905726, EPI_ISL_905727, EPI_ISL_905730, EPI_ISL_942978, EPI_ISL_942979, EPI_ISL_942980, EPI_ISL_942981, EPI_ISL_942982, EPI_ISL_942983, EPI_ISL_942984, EPI_ISL_942985, EPI_ISL_942986, EPI_ISL_942987, EPI_ISL_942988, EPI_ISL_942989, EPI_ISL_942990, EPI_ISL_942991, EPI_ISL_942992, EPI_ISL_942993, EPI_ISL_942994, EPI_ISL_942995, EPI_ISL_943001, EPI_ISL_943002, EPI_ISL_943003, EPI_ISL_943004, EPI_ISL_943005, EPI_ISL_943006, EPI_ISL_943007, EPI_ISL_943008, EPI_ISL_943009, EPI_ISL_943010, EPI_ISL_943011, EPI_ISL_943012, EPI_ISL_943013, EPI_ISL_943014, EPI_ISL_943015, EPI_ISL_943016, EPI_ISL_943017, EPI_ISL_943018, EPI_ISL_943019, EPI_ISL_943020, EPI_ISL_943021, EPI_ISL_943022, EPI_ISL_943023, EPI_ISL_943024, EPI_ISL_943025, EPI_ISL_943026, EPI_ISL_943027, EPI_ISL_943028, EPI_ISL_943029, EPI_ISL_943030, EPI_ISL_943031, EPI_ISL_943032, EPI_ISL_943033, EPI_ISL_943034, EPI_ISL_943035, EPI_ISL_943036, EPI_ISL_943037, EPI_ISL_943038, EPI_ISL_943039, EPI_ISL_943040, EPI_ISL_943041, EPI_ISL_943042, EPI_ISL_943043, EPI_ISL_943269, EPI_ISL_943272, EPI_ISL_943273, EPI_ISL_943274, EPI_ISL_943277, EPI_ISL_943278, EPI_ISL_943280, EPI_ISL_943281, EPI_ISL_943282, EPI_ISL_943283, EPI_ISL_943284, EPI_ISL_943286, EPI_ISL_943287, EPI_ISL_943291, EPI_ISL_943293, EPI_ISL_943298, EPI_ISL_943299, EPI_ISL_943300, EPI_ISL_943301, EPI_ISL_943302, EPI_ISL_943303, EPI_ISL_943308, EPI_ISL_943312, EPI_ISL_943313, EPI_ISL_943317, EPI_ISL_943319, EPI_ISL_943321, EPI_ISL_943322, EPI_ISL_943324, EPI_ISL_943325, EPI_ISL_943328, EPI_ISL_943335, EPI_ISL_943355, EPI_ISL_943357, EPI_ISL_943360, EPI_ISL_943361, EPI_ISL_943364, EPI_ISL_943365, EPI_ISL_943376, EPI_ISL_943381, EPI_ISL_943391, EPI_ISL_943404, EPI_ISL_943405, EPI_ISL_943409, EPI_ISL_943419, EPI_ISL_943427, EPI_ISL_943428, EPI_ISL_943429, EPI_ISL_943430, EPI_ISL_943432, EPI_ISL_943437, EPI_ISL_943440, EPI_ISL_943441, EPI_ISL_943442, EPI_ISL_943444, EPI_ISL_943449, EPI_ISL_943453, EPI_ISL_943458, EPI_ISL_943461, EPI_ISL_943462, EPI_ISL_943463, EPI_ISL_943464, EPI_ISL_943465, EPI_ISL_943468, EPI_ISL_943478, EPI_ISL_943479, EPI_ISL_943481, EPI_ISL_943482, EPI_ISL_943483, EPI_ISL_943484, EPI_ISL_943490, EPI_ISL_943491, EPI_ISL_943493, EPI_ISL_943496, EPI_ISL_943497, EPI_ISL_943504, EPI_ISL_943508, EPI_ISL_943518, EPI_ISL_943519, EPI_ISL_943532, EPI_ISL_943533, EPI_ISL_943539, EPI_ISL_1013619, EPI_ISL_1013620, EPI_ISL_1013621, EPI_ISL_1013622, EPI_ISL_1013623, EPI_ISL_1013624, EPI_ISL_1013625, EPI_ISL_1013626, EPI_ISL_1013627, EPI_ISL_1013628, EPI_ISL_1013629, EPI_ISL_1013630, EPI_ISL_1013631, EPI_ISL_1013632, EPI_ISL_1013633, EPI_ISL_1013634, EPI_ISL_1013635, EPI_ISL_1013636, EPI_ISL_1013637, EPI_ISL_1013638, EPI_ISL_1013639, EPI_ISL_1013640, EPI_ISL_1013641, EPI_ISL_1013642, EPI_ISL_1013643, EPI_ISL_1013644, EPI_ISL_1013645, EPI_ISL_1013646, EPI_ISL_1013647, EPI_ISL_1013648, EPI_ISL_1013649, EPI_ISL_1013650, EPI_ISL_1013651, EPI_ISL_1013652, EPI_ISL_1013653, EPI_ISL_1013654, EPI_ISL_1013655, EPI_ISL_1013656, EPI_ISL_1013657, EPI_ISL_1013658, EPI_ISL_1013659, EPI_ISL_1013660, EPI_ISL_1013661, EPI_ISL_1013662, EPI_ISL_1013663, EPI_ISL_1013664, EPI_ISL_1013665, EPI_ISL_1013666, EPI_ISL_1013667, EPI_ISL_1013668, EPI_ISL_1013669, EPI_ISL_1013670, EPI_ISL_1013671, EPI_ISL_1013672, EPI_ISL_1013673, EPI_ISL_1013674, EPI_ISL_1013675, EPI_ISL_1013676, EPI_ISL_1013677, EPI_ISL_1013678, EPI_ISL_1013679, EPI_ISL_1013680, EPI_ISL_1013681, EPI_ISL_1013685, EPI_ISL_1013686, EPI_ISL_1013687, EPI_ISL_1013688, EPI_ISL_1013689, EPI_ISL_1013695, EPI_ISL_1013697, EPI_ISL_1013698, EPI_ISL_1013699, EPI_ISL_1013700, EPI_ISL_1013701, EPI_ISL_1013702, EPI_ISL_1013703, EPI_ISL_1013704, EPI_ISL_1013705, EPI_ISL_1013706, EPI_ISL_1013707, EPI_ISL_1013708, EPI_ISL_1013709, EPI_ISL_1013710, EPI_ISL_1013711, EPI_ISL_1013712, EPI_ISL_1013713, EPI_ISL_1013714, EPI_ISL_1013715, EPI_ISL_1013716, EPI_ISL_1013717, EPI_ISL_1013718, EPI_ISL_1013719, EPI_ISL_1013720, EPI_ISL_1013721, EPI_ISL_1013722, EPI_ISL_1013723, EPI_ISL_1013724, EPI_ISL_1013725, EPI_ISL_1013726, EPI_ISL_1013727, EPI_ISL_1013728, EPI_ISL_1013729, EPI_ISL_1013730, EPI_ISL_1013731, EPI_ISL_1013732, EPI_ISL_1013733, EPI_ISL_1013734, EPI_ISL_1013735, EPI_ISL_1013736, EPI_ISL_1013737, EPI_ISL_1013738, EPI_ISL_1013739, EPI_ISL_1013740, EPI_ISL_1013741, EPI_ISL_1013742, EPI_ISL_1013743, EPI_ISL_1013744, EPI_ISL_1013745, EPI_ISL_1013746, EPI_ISL_1013747, EPI_ISL_1013748, EPI_ISL_1013749, EPI_ISL_1013750, EPI_ISL_1013751, EPI_ISL_1013752, EPI_ISL_1013753, EPI_ISL_1013754, EPI_ISL_1013755, EPI_ISL_1013756, EPI_ISL_1013757, EPI_ISL_1013758, EPI_ISL_1013759, EPI_ISL_1013760, EPI_ISL_1013761, EPI_ISL_1013762, EPI_ISL_1013763, EPI_ISL_1013764, EPI_ISL_1013765, EPI_ISL_1013766, EPI_ISL_1013767, EPI_ISL_1013768, EPI_ISL_1013769, EPI_ISL_1013770, EPI_ISL_1014241, EPI_ISL_1014242, EPI_ISL_1014243, EPI_ISL_1014244, EPI_ISL_1014245, EPI_ISL_1014246, EPI_ISL_1014247, EPI_ISL_1014248, EPI_ISL_1014249, EPI_ISL_1014250, EPI_ISL_1014251, EPI_ISL_1014252, EPI_ISL_1014253, EPI_ISL_1014254, EPI_ISL_1014255, EPI_ISL_1014256, EPI_ISL_1014257, EPI_ISL_1014258, EPI_ISL_1014259, EPI_ISL_1014260, EPI_ISL_1014261, EPI_ISL_1014262, EPI_ISL_1014263, EPI_ISL_1014264, EPI_ISL_1014265, EPI_ISL_1014266, EPI_ISL_1014267, EPI_ISL_1014268, EPI_ISL_1014272, EPI_ISL_1014273, EPI_ISL_1014274, EPI_ISL_1014275, EPI_ISL_1014276, EPI_ISL_1014277, EPI_ISL_1014285, EPI_ISL_1014286, EPI_ISL_1014287, EPI_ISL_1014288, EPI_ISL_1014289, EPI_ISL_1014290, EPI_ISL_1014291, EPI_ISL_1014292, EPI_ISL_1014293, EPI_ISL_1014294, EPI_ISL_1014295, EPI_ISL_1014296, EPI_ISL_1014297, EPI_ISL_1014298, EPI_ISL_1014299, EPI_ISL_1014300, EPI_ISL_1014301, EPI_ISL_1014302, EPI_ISL_1014303, EPI_ISL_1014304, EPI_ISL_1014305, EPI_ISL_1014306, EPI_ISL_1014307, EPI_ISL_1014308, EPI_ISL_1014309, EPI_ISL_1014310, EPI_ISL_1014311, EPI_ISL_1014312, EPI_ISL_1014313, EPI_ISL_1014314, EPI_ISL_1014315, EPI_ISL_1014316, EPI_ISL_1014317, EPI_ISL_1014318, EPI_ISL_1014319, EPI_ISL_1014320, EPI_ISL_1014321, EPI_ISL_1014322, EPI_ISL_1014323, EPI_ISL_1014324, EPI_ISL_1014325, EPI_ISL_1014326, EPI_ISL_1014327, EPI_ISL_1014328, EPI_ISL_1020302, EPI_ISL_1034927, EPI_ISL_1034928, EPI_ISL_1034929, EPI_ISL_1034930, EPI_ISL_1034931, EPI_ISL_1034932, EPI_ISL_1034933, EPI_ISL_1034934, EPI_ISL_1034935, EPI_ISL_1034936, EPI_ISL_1034937, EPI_ISL_1034938, EPI_ISL_1034939, EPI_ISL_1034940, EPI_ISL_1034941, EPI_ISL_1034942, EPI_ISL_1034943, EPI_ISL_1034944, EPI_ISL_1034945, EPI_ISL_1034946, EPI_ISL_1034947, EPI_ISL_1034948, EPI_ISL_1034949, EPI_ISL_1034950, EPI_ISL_1034951, EPI_ISL_1034952, EPI_ISL_1034953, EPI_ISL_1034954, EPI_ISL_1034955, EPI_ISL_1034956, EPI_ISL_1034957, EPI_ISL_1034958, EPI_ISL_1034959, EPI_ISL_1034960, EPI_ISL_1034961, EPI_ISL_1034962, EPI_ISL_1034963, EPI_ISL_1034964, EPI_ISL_1034965, EPI_ISL_1034966, EPI_ISL_1034967, EPI_ISL_1034968, EPI_ISL_1034969, EPI_ISL_1034970, EPI_ISL_1034971, EPI_ISL_1034972, EPI_ISL_1034973, EPI_ISL_1034974, EPI_ISL_1034975, EPI_ISL_1034976, EPI_ISL_1034977, EPI_ISL_1034978, EPI_ISL_1034979, EPI_ISL_1034980, EPI_ISL_1034981, EPI_ISL_1034982, EPI_ISL_1034983, EPI_ISL_1034984, EPI_ISL_1034985, EPI_ISL_1034986, EPI_ISL_1034987, EPI_ISL_1034988, EPI_ISL_1034989, EPI_ISL_1034990, EPI_ISL_1034991, EPI_ISL_1034992, EPI_ISL_1034993, EPI_ISL_1034994, EPI_ISL_1034995, EPI_ISL_1034996, EPI_ISL_1034997, EPI_ISL_1034998, EPI_ISL_1034999, EPI_ISL_1035000, EPI_ISL_1035001, EPI_ISL_1035002, EPI_ISL_1035003, EPI_ISL_1035004, EPI_ISL_1035005, EPI_ISL_1035006, EPI_ISL_1035007, EPI_ISL_1035008, EPI_ISL_1035009, EPI_ISL_1035010, EPI_ISL_1035011, EPI_ISL_1035012, EPI_ISL_1035013, EPI_ISL_1035014, EPI_ISL_1035015, EPI_ISL_1035016, EPI_ISL_1035017, EPI_ISL_1035018, EPI_ISL_1035019, EPI_ISL_1035020, EPI_ISL_1035021, EPI_ISL_1035022, EPI_ISL_1035023, EPI_ISL_1035024, EPI_ISL_1035025, EPI_ISL_1035026, EPI_ISL_1035027, EPI_ISL_1035028, EPI_ISL_1035029, EPI_ISL_1035030, EPI_ISL_1035031, EPI_ISL_1035032, EPI_ISL_1035033, EPI_ISL_1035034, EPI_ISL_1035035, EPI_ISL_1035036, EPI_ISL_1035037, EPI_ISL_1035038, EPI_ISL_1035039, EPI_ISL_1035040, EPI_ISL_1035041, EPI_ISL_1035042, EPI_ISL_1035043, EPI_ISL_1035044, EPI_ISL_1035045, EPI_ISL_1035046, EPI_ISL_1035047, EPI_ISL_1035048, EPI_ISL_1035049, EPI_ISL_1035050, EPI_ISL_1035051, EPI_ISL_1035052, EPI_ISL_1035053, EPI_ISL_1035054, EPI_ISL_1035055, EPI_ISL_1035056, EPI_ISL_1035057, EPI_ISL_1035058, EPI_ISL_1035059, EPI_ISL_1035060, EPI_ISL_1035068, EPI_ISL_1035069, EPI_ISL_1035070, EPI_ISL_1035071, EPI_ISL_1035072, EPI_ISL_1035073, EPI_ISL_1035074, EPI_ISL_1035075, EPI_ISL_1035076, EPI_ISL_1035077, EPI_ISL_1035078, EPI_ISL_1035101, EPI_ISL_1035102, EPI_ISL_1035103, EPI_ISL_1035104, EPI_ISL_1035105, EPI_ISL_1035106, EPI_ISL_1035107, EPI_ISL_1035108, EPI_ISL_1035109, EPI_ISL_1035110, EPI_ISL_1035111, EPI_ISL_1035112, EPI_ISL_1035113, EPI_ISL_1035114, EPI_ISL_1035115, EPI_ISL_1035116, EPI_ISL_1035117, EPI_ISL_1035118, EPI_ISL_1035119, EPI_ISL_1035120, EPI_ISL_1035121, EPI_ISL_1035122, EPI_ISL_1035123, EPI_ISL_1035124, EPI_ISL_1035125, EPI_ISL_1035126, EPI_ISL_1035127, EPI_ISL_1035128, EPI_ISL_1035129, EPI_ISL_1035130, EPI_ISL_1035131, EPI_ISL_1035132, EPI_ISL_1035133, EPI_ISL_1035134, EPI_ISL_1035135, EPI_ISL_1035136, EPI_ISL_1035137, EPI_ISL_1035138, EPI_ISL_1035139, EPI_ISL_1035140, EPI_ISL_1035141, EPI_ISL_1035142, EPI_ISL_1035143, EPI_ISL_1035144, EPI_ISL_1035145, EPI_ISL_1035146, EPI_ISL_1035147, EPI_ISL_1035148, EPI_ISL_1035149, EPI_ISL_1035150, EPI_ISL_1035151, EPI_ISL_1035152, EPI_ISL_1035153, EPI_ISL_1035154, EPI_ISL_1035155, EPI_ISL_1035156, EPI_ISL_1035157, EPI_ISL_1035158, EPI_ISL_1035159, EPI_ISL_1035160, EPI_ISL_1035161, EPI_ISL_1035162, EPI_ISL_1035163, EPI_ISL_1035164, EPI_ISL_1035165, EPI_ISL_1035166, EPI_ISL_1035167, EPI_ISL_1035168, EPI_ISL_1035169, EPI_ISL_1035170, EPI_ISL_1035171, EPI_ISL_1035172, EPI_ISL_1035173, EPI_ISL_1035174, EPI_ISL_1035175, EPI_ISL_1035176, EPI_ISL_1035177, EPI_ISL_1035178, EPI_ISL_1035179, EPI_ISL_1035180, EPI_ISL_1035181, EPI_ISL_1035182, EPI_ISL_1035183, EPI_ISL_1035184, EPI_ISL_1035185, EPI_ISL_1035186, EPI_ISL_1035187, EPI_ISL_1035188, EPI_ISL_1035189, EPI_ISL_1035190, EPI_ISL_1035191, EPI_ISL_1035192, EPI_ISL_1035193, EPI_ISL_1035194, EPI_ISL_1035195, EPI_ISL_1035196, EPI_ISL_1035197, EPI_ISL_1059903, EPI_ISL_1059904, EPI_ISL_1089435, EPI_ISL_1089436, EPI_ISL_1089437, EPI_ISL_1089442, EPI_ISL_1089443, EPI_ISL_1089444, EPI_ISL_1089445, EPI_ISL_1089446, EPI_ISL_1089447, EPI_ISL_1089448, EPI_ISL_1089449, EPI_ISL_1089450, EPI_ISL_1089451, EPI_ISL_1089452, EPI_ISL_1089453, EPI_ISL_1089454, EPI_ISL_1089455, EPI_ISL_1089456, EPI_ISL_1089457, EPI_ISL_1089458, EPI_ISL_1089459, EPI_ISL_1089460, EPI_ISL_1089461, EPI_ISL_1089462, EPI_ISL_1089463, EPI_ISL_1089464, EPI_ISL_1089465, EPI_ISL_1089466, EPI_ISL_1089467, EPI_ISL_1089468, EPI_ISL_1089469, EPI_ISL_1089470, EPI_ISL_1089471, EPI_ISL_1089472, EPI_ISL_1089473, EPI_ISL_1089474, EPI_ISL_1089475, EPI_ISL_1089476, EPI_ISL_1089477, EPI_ISL_1089478, EPI_ISL_1089479, EPI_ISL_1089480, EPI_ISL_1089481, EPI_ISL_1089482, EPI_ISL_1089483, EPI_ISL_1089484, EPI_ISL_1089485, EPI_ISL_1089486, EPI_ISL_1089487, EPI_ISL_1089488, EPI_ISL_1089489, EPI_ISL_1089490, EPI_ISL_1089492, EPI_ISL_1089493, EPI_ISL_1089494, EPI_ISL_1089495, EPI_ISL_1089496, EPI_ISL_1089497, EPI_ISL_1089498, EPI_ISL_1089499, EPI_ISL_1089500, EPI_ISL_1089501, EPI_ISL_1089502, EPI_ISL_1089503, EPI_ISL_1089504, EPI_ISL_1089505, EPI_ISL_1089506, EPI_ISL_1089507, EPI_ISL_1089508, EPI_ISL_1089509, EPI_ISL_1089510, EPI_ISL_1089511, EPI_ISL_1089512, EPI_ISL_1089514, EPI_ISL_1089515, EPI_ISL_1089516, EPI_ISL_1089517, EPI_ISL_1089518, EPI_ISL_1089519, EPI_ISL_1089520, EPI_ISL_1089521, EPI_ISL_1089522, EPI_ISL_1089523, EPI_ISL_1089524, EPI_ISL_1089525, EPI_ISL_1089526, EPI_ISL_1089527, EPI_ISL_1089528, EPI_ISL_1089529, EPI_ISL_1089530, EPI_ISL_1089531, EPI_ISL_1089532, EPI_ISL_1089533, EPI_ISL_1089534, EPI_ISL_1089535, EPI_ISL_1089536, EPI_ISL_1089537, EPI_ISL_1089538, EPI_ISL_1089539, EPI_ISL_1089540, EPI_ISL_1089541, EPI_ISL_1089542, EPI_ISL_1089543, EPI_ISL_1089544, EPI_ISL_1089545, EPI_ISL_1089546, EPI_ISL_1089547, EPI_ISL_1089548, EPI_ISL_1089549, EPI_ISL_1089550, EPI_ISL_1089551, EPI_ISL_1089552, EPI_ISL_1089553, EPI_ISL_1089554, EPI_ISL_1089555, EPI_ISL_1089556, EPI_ISL_1089557, EPI_ISL_1089558, EPI_ISL_1089559, EPI_ISL_1089560, EPI_ISL_1089561, EPI_ISL_1089562, EPI_ISL_1089563, EPI_ISL_1089564, EPI_ISL_1089565, EPI_ISL_1089566, EPI_ISL_1089567, EPI_ISL_1089568, EPI_ISL_1089569, EPI_ISL_1089570, EPI_ISL_1089571, EPI_ISL_1089572, EPI_ISL_1089573, EPI_ISL_1089574, EPI_ISL_1089575, EPI_ISL_1089576, EPI_ISL_1089577, EPI_ISL_1089578, EPI_ISL_1089579, EPI_ISL_1089580, EPI_ISL_1089581, EPI_ISL_1089582, EPI_ISL_1089593, EPI_ISL_1089594, EPI_ISL_1089595, EPI_ISL_1089596, EPI_ISL_1089597, EPI_ISL_1089598, EPI_ISL_1089599, EPI_ISL_1089600, EPI_ISL_1089601, EPI_ISL_1089602, EPI_ISL_1089603, EPI_ISL_1089608, EPI_ISL_1089609, EPI_ISL_1089628, EPI_ISL_1089629, EPI_ISL_1089631, EPI_ISL_1089632, EPI_ISL_1089633, EPI_ISL_1089634, EPI_ISL_1089635, EPI_ISL_1089636, EPI_ISL_1089637, EPI_ISL_1089638, EPI_ISL_1089639, EPI_ISL_1089640, EPI_ISL_1089641, EPI_ISL_1089642, EPI_ISL_1089643, EPI_ISL_1089644, EPI_ISL_1089645, EPI_ISL_1089646, EPI_ISL_1089647, EPI_ISL_1089648, EPI_ISL_1089649, EPI_ISL_1089650, EPI_ISL_1089651, EPI_ISL_1089652, EPI_ISL_1089653, EPI_ISL_1089654, EPI_ISL_1089655, EPI_ISL_1089656, EPI_ISL_1089657, EPI_ISL_1089658, EPI_ISL_1089659, EPI_ISL_1089660, EPI_ISL_1089661, EPI_ISL_1089662, EPI_ISL_1089663, EPI_ISL_1089664, EPI_ISL_1089665, EPI_ISL_1089666, EPI_ISL_1089667, EPI_ISL_1089668, EPI_ISL_1089669, EPI_ISL_1089670, EPI_ISL_1089671, EPI_ISL_1089672, EPI_ISL_1089673, EPI_ISL_1089674, EPI_ISL_1089675, EPI_ISL_1089676, EPI_ISL_1089677, EPI_ISL_1089678, EPI_ISL_1089679, EPI_ISL_1089680, EPI_ISL_1089681, EPI_ISL_1089682, EPI_ISL_1089683, EPI_ISL_1089684, EPI_ISL_1089685, EPI_ISL_1089686, EPI_ISL_1089687, EPI_ISL_1089688, EPI_ISL_1089689, EPI_ISL_1089690, EPI_ISL_1089691, EPI_ISL_1089692, EPI_ISL_1089693, EPI_ISL_1089694, EPI_ISL_1089695, EPI_ISL_1089696, EPI_ISL_1089697, EPI_ISL_1089698, EPI_ISL_1089699, EPI_ISL_1089700, EPI_ISL_1089701, EPI_ISL_1089702, EPI_ISL_1089703, EPI_ISL_1089704, EPI_ISL_1089705, EPI_ISL_1089706, EPI_ISL_1089707, EPI_ISL_1089708, EPI_ISL_1089709, EPI_ISL_1089710, EPI_ISL_1089711, EPI_ISL_1089712, EPI_ISL_1089713, EPI_ISL_1089714, EPI_ISL_1089715, EPI_ISL_1089716, EPI_ISL_1089717, EPI_ISL_1089718, EPI_ISL_1089719, EPI_ISL_1089720, EPI_ISL_1089721, EPI_ISL_1089722, EPI_ISL_1089723, EPI_ISL_1089724, EPI_ISL_1089725, EPI_ISL_1089726, EPI_ISL_1089727, EPI_ISL_1089728, EPI_ISL_1089729, EPI_ISL_1089730, EPI_ISL_1089731, EPI_ISL_1089732, EPI_ISL_1089733, EPI_ISL_1089734, EPI_ISL_1089735, EPI_ISL_1089736, EPI_ISL_1089737, EPI_ISL_1089738, EPI_ISL_1089739, EPI_ISL_1089740, EPI_ISL_1089741, EPI_ISL_1089742, EPI_ISL_1089743, EPI_ISL_1089744, EPI_ISL_1089745, EPI_ISL_1089746, EPI_ISL_1089747, EPI_ISL_1089748, EPI_ISL_1089749, EPI_ISL_1089750, EPI_ISL_1089751, EPI_ISL_1089752, EPI_ISL_1089753, EPI_ISL_1089754, EPI_ISL_1089755, EPI_ISL_1089756, EPI_ISL_1089757, EPI_ISL_1089758, EPI_ISL_1089759, EPI_ISL_1089760, EPI_ISL_1089761, EPI_ISL_1089762, EPI_ISL_1089763, EPI_ISL_1089764, EPI_ISL_1089765, EPI_ISL_1089766, EPI_ISL_1089767, EPI_ISL_1089768, EPI_ISL_1089769, EPI_ISL_1089770, EPI_ISL_1089771, EPI_ISL_1089772, EPI_ISL_1089805, EPI_ISL_1165144, EPI_ISL_1165145, EPI_ISL_1165146, EPI_ISL_1165147, EPI_ISL_1165148, EPI_ISL_1165149, EPI_ISL_1165150, EPI_ISL_1165151, EPI_ISL_1165152, EPI_ISL_1165153, EPI_ISL_1165154, EPI_ISL_1165155, EPI_ISL_1165156, EPI_ISL_1165157, EPI_ISL_1165158, EPI_ISL_1165159, EPI_ISL_1165160, EPI_ISL_1165161, EPI_ISL_1165162, EPI_ISL_1165163, EPI_ISL_1165164, EPI_ISL_1165165, EPI_ISL_1165166, EPI_ISL_1165167, EPI_ISL_1165168, EPI_ISL_1165169, EPI_ISL_1165170, EPI_ISL_1165171, EPI_ISL_1165172, EPI_ISL_1165173, EPI_ISL_1165174, EPI_ISL_1165175, EPI_ISL_1165176, EPI_ISL_1165177, EPI_ISL_1165178, EPI_ISL_1165179, EPI_ISL_1165180, EPI_ISL_1165181, EPI_ISL_1165182, EPI_ISL_1165183, EPI_ISL_1165184, EPI_ISL_1165185, EPI_ISL_1165186, EPI_ISL_1165187, EPI_ISL_1165188, EPI_ISL_1165189, EPI_ISL_1165190, EPI_ISL_1165191, EPI_ISL_1165192, EPI_ISL_1165193, EPI_ISL_1165194, EPI_ISL_1165195, EPI_ISL_1165196, EPI_ISL_1165197, EPI_ISL_1165203, EPI_ISL_1165204, EPI_ISL_1165205, EPI_ISL_1165206, EPI_ISL_1165207, EPI_ISL_1165208, EPI_ISL_1165209, EPI_ISL_1165210, EPI_ISL_1165211, EPI_ISL_1165212, EPI_ISL_1165213, EPI_ISL_1165214, EPI_ISL_1165215, EPI_ISL_1165216, EPI_ISL_1165217, EPI_ISL_1165218, EPI_ISL_1165219, EPI_ISL_1165220, EPI_ISL_1165221, EPI_ISL_1165222, EPI_ISL_1165223, EPI_ISL_1165224, EPI_ISL_1165225, EPI_ISL_1165226, EPI_ISL_1165227, EPI_ISL_1165228, EPI_ISL_1165229, EPI_ISL_1165230, EPI_ISL_1165231, EPI_ISL_1165232, EPI_ISL_1165233, EPI_ISL_1165234, EPI_ISL_1165235, EPI_ISL_1165236, EPI_ISL_1165237, EPI_ISL_1165238, EPI_ISL_1165239, EPI_ISL_1165240, EPI_ISL_1165241, EPI_ISL_1165242, EPI_ISL_1165243, EPI_ISL_1165244, EPI_ISL_1165245, EPI_ISL_1165246, EPI_ISL_1165247, EPI_ISL_1165248, EPI_ISL_1165249, EPI_ISL_1165250, EPI_ISL_1165251, EPI_ISL_1165252, EPI_ISL_1165253, EPI_ISL_1165254, EPI_ISL_1165255, EPI_ISL_1165256, EPI_ISL_1165257, EPI_ISL_1165258, EPI_ISL_1165259, EPI_ISL_1165260, EPI_ISL_1165261, EPI_ISL_1165262, EPI_ISL_1165263, EPI_ISL_1165264, EPI_ISL_1165265, EPI_ISL_1165266, EPI_ISL_1165267, EPI_ISL_1165268, EPI_ISL_1165269, EPI_ISL_1165270, EPI_ISL_1165271, EPI_ISL_1165272, EPI_ISL_1165273, EPI_ISL_1165274, EPI_ISL_1165275, EPI_ISL_1165276, EPI_ISL_1165277, EPI_ISL_1165278, EPI_ISL_1165279, EPI_ISL_1165280, EPI_ISL_1165281, EPI_ISL_1165282, EPI_ISL_1165283, EPI_ISL_1165284, EPI_ISL_1165285, EPI_ISL_1165286, EPI_ISL_1165287, EPI_ISL_1165288, EPI_ISL_1165289, EPI_ISL_1165290, EPI_ISL_1165291, EPI_ISL_1165292, EPI_ISL_1165293, EPI_ISL_1165294, EPI_ISL_1165295, EPI_ISL_1165296, EPI_ISL_1165297, EPI_ISL_1165298, EPI_ISL_1165299, EPI_ISL_1165300, EPI_ISL_1165301, EPI_ISL_1165302, EPI_ISL_1165303, EPI_ISL_1165304, EPI_ISL_1165305, EPI_ISL_1165306, EPI_ISL_1165307, EPI_ISL_1165308, EPI_ISL_1165309, EPI_ISL_1165310, EPI_ISL_1165311, EPI_ISL_1165312, EPI_ISL_1165314, EPI_ISL_1165315, EPI_ISL_1165316, EPI_ISL_1165317, EPI_ISL_1165318, EPI_ISL_1165319, EPI_ISL_1165320, EPI_ISL_1165321, EPI_ISL_1165322, EPI_ISL_1165323, EPI_ISL_1165324, EPI_ISL_1165325, EPI_ISL_1165326, EPI_ISL_1165327, EPI_ISL_1165328, EPI_ISL_1165329, EPI_ISL_1165330, EPI_ISL_1165331, EPI_ISL_1165332, EPI_ISL_1165333, EPI_ISL_1165334, EPI_ISL_1165335, EPI_ISL_1165336, EPI_ISL_1165337, EPI_ISL_1165338, EPI_ISL_1165339, EPI_ISL_1165340, EPI_ISL_1165341, EPI_ISL_1165342, EPI_ISL_1165343, EPI_ISL_1165344, EPI_ISL_1165345, EPI_ISL_1165346, EPI_ISL_1165347, EPI_ISL_1165348, EPI_ISL_1165349, EPI_ISL_1165350, EPI_ISL_1165351, EPI_ISL_1165352, EPI_ISL_1165353, EPI_ISL_1165354, EPI_ISL_1165355, EPI_ISL_1165356, EPI_ISL_1165357, EPI_ISL_1165358, EPI_ISL_1165359, EPI_ISL_1165360, EPI_ISL_1165361, EPI_ISL_1165362, EPI_ISL_1165363, EPI_ISL_1165364, EPI_ISL_1165365, EPI_ISL_1165366, EPI_ISL_1165367, EPI_ISL_1165368, EPI_ISL_1165372, EPI_ISL_1165374, EPI_ISL_1165377, EPI_ISL_1165383, EPI_ISL_1165385, EPI_ISL_1165386, EPI_ISL_1165387, EPI_ISL_1165388, EPI_ISL_1165389, EPI_ISL_1165390, EPI_ISL_1165391, EPI_ISL_1165392, EPI_ISL_1165393, EPI_ISL_1165394, EPI_ISL_1165395, EPI_ISL_1165396, EPI_ISL_1165397, EPI_ISL_1165398,			

EPI_ISL_1165399, EPI_ISL_1165400, EPI_ISL_1165401, EPI_ISL_1165402, EPI_ISL_1165403, EPI_ISL_1165404, EPI_ISL_1165405, EPI_ISL_1165406, EPI_ISL_1165407, EPI_ISL_1165408, EPI_ISL_1165409, EPI_ISL_1165410, EPI_ISL_1165411, EPI_ISL_1165412, EPI_ISL_1165413, EPI_ISL_1165414, EPI_ISL_1165415, EPI_ISL_1165416, EPI_ISL_1165417, EPI_ISL_1165418, EPI_ISL_1165419, EPI_ISL_1165420, EPI_ISL_1165421, EPI_ISL_1165422, EPI_ISL_1165423, EPI_ISL_1165424, EPI_ISL_1165425, EPI_ISL_1165426, EPI_ISL_1165427, EPI_ISL_1165428, EPI_ISL_1165429, EPI_ISL_1165430, EPI_ISL_1165431, EPI_ISL_1165432, EPI_ISL_1165433, EPI_ISL_1165434, EPI_ISL_1165435, EPI_ISL_1165436, EPI_ISL_1165437, EPI_ISL_1165438, EPI_ISL_1165439, EPI_ISL_1165440, EPI_ISL_1165441, EPI_ISL_1165443, EPI_ISL_1165444, EPI_ISL_1165445, EPI_ISL_1165446, EPI_ISL_1165447, EPI_ISL_1165448, EPI_ISL_1165449, EPI_ISL_1165450, EPI_ISL_1165451, EPI_ISL_1165452, EPI_ISL_1165453, EPI_ISL_1165454, EPI_ISL_1165455, EPI_ISL_1165456, EPI_ISL_1165457, EPI_ISL_1165458, EPI_ISL_1165459, EPI_ISL_1165460, EPI_ISL_1165461, EPI_ISL_1165462, EPI_ISL_1165463, EPI_ISL_1165464, EPI_ISL_1165465, EPI_ISL_1165466, EPI_ISL_1165467, EPI_ISL_1165468, EPI_ISL_1165469, EPI_ISL_1165470, EPI_ISL_1165471, EPI_ISL_1165472, EPI_ISL_1165473, EPI_ISL_1165474, EPI_ISL_1165475, EPI_ISL_1165476, EPI_ISL_1165477, EPI_ISL_1165478, EPI_ISL_1165479, EPI_ISL_1165480, EPI_ISL_1165481, EPI_ISL_1165482, EPI_ISL_1165483, EPI_ISL_1165484, EPI_ISL_1165485, EPI_ISL_1165486, EPI_ISL_1165487, EPI_ISL_1165488, EPI_ISL_1165489, EPI_ISL_1165490, EPI_ISL_1165491, EPI_ISL_1165492, EPI_ISL_1165493, EPI_ISL_1165494, EPI_ISL_1165495, EPI_ISL_1165496, EPI_ISL_1165497, EPI_ISL_1165498, EPI_ISL_1165499, EPI_ISL_1165500, EPI_ISL_1165501, EPI_ISL_1165502, EPI_ISL_1165503, EPI_ISL_1165504, EPI_ISL_1165506, EPI_ISL_1165507, EPI_ISL_1165534, EPI_ISL_1165535, EPI_ISL_1165536, EPI_ISL_1165537, EPI_ISL_1165538, EPI_ISL_1165539, EPI_ISL_1165540, EPI_ISL_1165541, EPI_ISL_1165542, EPI_ISL_1165543, EPI_ISL_1165544, EPI_ISL_1165545, EPI_ISL_1165546, EPI_ISL_1165547, EPI_ISL_1165548, EPI_ISL_1165549, EPI_ISL_1165550, EPI_ISL_1165551, EPI_ISL_1165563, EPI_ISL_1216241, EPI_ISL_1216261, EPI_ISL_1216267, EPI_ISL_1216283, EPI_ISL_1216285, EPI_ISL_1216288, EPI_ISL_1216296, EPI_ISL_1216307, EPI_ISL_1216319, EPI_ISL_1216326, EPI_ISL_1216334, EPI_ISL_1216341, EPI_ISL_1216345, EPI_ISL_1216350, EPI_ISL_1232243, EPI_ISL_1232250, EPI_ISL_1232251, EPI_ISL_1232254, EPI_ISL_1232256, EPI_ISL_1232261, EPI_ISL_1232262, EPI_ISL_1232263, EPI_ISL_1232266, EPI_ISL_1232274, EPI_ISL_1232275, EPI_ISL_1232277, EPI_ISL_1232278, EPI_ISL_1232285, EPI_ISL_1232286, EPI_ISL_1232287, EPI_ISL_1232288, EPI_ISL_1232289, EPI_ISL_1232297, EPI_ISL_1232298, EPI_ISL_1232300, EPI_ISL_1232308, EPI_ISL_1232320, EPI_ISL_1232323, EPI_ISL_1232324, EPI_ISL_1232331, EPI_ISL_1232334, EPI_ISL_1232337, EPI_ISL_1232339, EPI_ISL_1232341, EPI_ISL_1232345, EPI_ISL_1232346, EPI_ISL_1232348, EPI_ISL_1232350, EPI_ISL_1232353, EPI_ISL_1232357, EPI_ISL_1232358, EPI_ISL_1232359, EPI_ISL_1232361, EPI_ISL_1232363, EPI_ISL_1232364, EPI_ISL_1232365, EPI_ISL_1232366, EPI_ISL_1232367, EPI_ISL_1232368, EPI_ISL_1232369, EPI_ISL_1232372, EPI_ISL_1232374, EPI_ISL_1232378, EPI_ISL_1232380, EPI_ISL_1232381, EPI_ISL_1232385, EPI_ISL_1232386, EPI_ISL_1232387, EPI_ISL_1232388, EPI_ISL_1232389, EPI_ISL_1232390, EPI_ISL_1232391, EPI_ISL_1232395, EPI_ISL_1232396, EPI_ISL_1232397, EPI_ISL_1232400, EPI_ISL_1232404, EPI_ISL_1232405, EPI_ISL_1232406, EPI_ISL_1232407, EPI_ISL_1232416, EPI_ISL_1232417, EPI_ISL_1232423, EPI_ISL_1232425, EPI_ISL_1232428, EPI_ISL_1232430, EPI_ISL_1232434, EPI_ISL_1232436, EPI_ISL_1232437, EPI_ISL_1232440, EPI_ISL_1232441, EPI_ISL_1232444, EPI_ISL_1232447, EPI_ISL_1232448, EPI_ISL_1232449, EPI_ISL_1232450, EPI_ISL_1232451, EPI_ISL_1232455, EPI_ISL_1232456, EPI_ISL_1232457, EPI_ISL_1232458, EPI_ISL_1232459, EPI_ISL_1232460, EPI_ISL_1232463, EPI_ISL_1232464, EPI_ISL_1232465, EPI_ISL_1232469, EPI_ISL_1232472, EPI_ISL_1232473, EPI_ISL_1232475, EPI_ISL_1232476, EPI_ISL_1232477, EPI_ISL_1232478, EPI_ISL_1232480, EPI_ISL_1232484, EPI_ISL_1232489, EPI_ISL_1232490, EPI_ISL_1232491, EPI_ISL_1232496, EPI_ISL_1232497, EPI_ISL_1232498, EPI_ISL_1232499, EPI_ISL_1232500, EPI_ISL_1232502, EPI_ISL_1232503, EPI_ISL_1232506, EPI_ISL_1232507, EPI_ISL_1232508, EPI_ISL_1232511, EPI_ISL_1232512, EPI_ISL_1232516, EPI_ISL_1232517, EPI_ISL_1232518, EPI_ISL_1232519, EPI_ISL_1232521, EPI_ISL_1232522, EPI_ISL_1232523, EPI_ISL_1232524, EPI_ISL_1232526, EPI_ISL_1232527, EPI_ISL_1232529, EPI_ISL_1232531, EPI_ISL_1232539, EPI_ISL_1232541, EPI_ISL_1232544, EPI_ISL_1232551, EPI_ISL_1232556, EPI_ISL_1232563, EPI_ISL_1232573, EPI_ISL_1232575, EPI_ISL_1232585, EPI_ISL_1232589, EPI_ISL_1232590, EPI_ISL_1232594, EPI_ISL_1232595, EPI_ISL_1232598, EPI_ISL_1232599, EPI_ISL_1232605, EPI_ISL_1232610, EPI_ISL_1232611, EPI_ISL_1232617, EPI_ISL_1232618, EPI_ISL_1232622, EPI_ISL_1232623, EPI_ISL_1232627, EPI_ISL_1232628, EPI_ISL_1232629, EPI_ISL_1232634, EPI_ISL_1232636, EPI_ISL_1232642, EPI_ISL_1232643, EPI_ISL_1232644, EPI_ISL_1232645, EPI_ISL_1232646, EPI_ISL_1232647, EPI_ISL_1232652, EPI_ISL_1232653, EPI_ISL_1232658, EPI_ISL_1232661, EPI_ISL_1232662, EPI_ISL_1232667, EPI_ISL_1232676, EPI_ISL_1232679, EPI_ISL_1232680, EPI_ISL_1232682, EPI_ISL_1232684, EPI_ISL_1232690, EPI_ISL_1232700, EPI_ISL_1232710, EPI_ISL_1232713, EPI_ISL_1232718, EPI_ISL_1232724, EPI_ISL_1232729, EPI_ISL_1232740, EPI_ISL_1232767, EPI_ISL_1232768, EPI_ISL_1232769, EPI_ISL_1232770, EPI_ISL_1232772, EPI_ISL_1232773, EPI_ISL_1232774, EPI_ISL_1232778, EPI_ISL_1232780, EPI_ISL_1232783, EPI_ISL_1232784, EPI_ISL_1232785, EPI_ISL_1232791, EPI_ISL_1232794, EPI_ISL_1232797, EPI_ISL_1232798, EPI_ISL_1232800, EPI_ISL_1232801, EPI_ISL_1232803, EPI_ISL_1232804, EPI_ISL_1232805, EPI_ISL_1232806, EPI_ISL_1232811, EPI_ISL_1232813, EPI_ISL_1232819, EPI_ISL_1232821, EPI_ISL_1232824, EPI_ISL_1232825, EPI_ISL_1232826, EPI_ISL_1232828, EPI_ISL_1232829, EPI_ISL_1232830, EPI_ISL_1232833, EPI_ISL_1232834, EPI_ISL_1232838, EPI_ISL_1232843, EPI_ISL_1232844, EPI_ISL_1232846, EPI_ISL_1232850, EPI_ISL_1232851, EPI_ISL_1232854, EPI_ISL_1232855, EPI_ISL_1232858, EPI_ISL_1232859, EPI_ISL_1232861, EPI_ISL_1232866, EPI_ISL_1232867, EPI_ISL_1232871, EPI_ISL_1232872, EPI_ISL_1232873, EPI_ISL_1232875, EPI_ISL_1232878, EPI_ISL_1232880, EPI_ISL_1232884, EPI_ISL_1232886, EPI_ISL_1232892, EPI_ISL_1232893, EPI_ISL_1232900, EPI_ISL_1232902, EPI_ISL_1232918, EPI_ISL_1232923, EPI_ISL_1232924, EPI_ISL_1232935, EPI_ISL_1232941, EPI_ISL_1232942, EPI_ISL_1232947, EPI_ISL_1232953, EPI_ISL_1232959, EPI_ISL_1232960, EPI_ISL_1232965, EPI_ISL_1232966, EPI_ISL_1232976, EPI_ISL_1232982, EPI_ISL_1232983, EPI_ISL_1232994, EPI_ISL_1233006, EPI_ISL_1287791, EPI_ISL_1287792, EPI_ISL_1287794, EPI_ISL_1287798, EPI_ISL_1287800, EPI_ISL_1287801, EPI_ISL_1287802, EPI_ISL_1287804, EPI_ISL_1287805, EPI_ISL_1287811, EPI_ISL_1287818, EPI_ISL_1287823, EPI_ISL_1287830, EPI_ISL_1287878, EPI_ISL_1287879, EPI_ISL_1287883, EPI_ISL_1287885, EPI_ISL_1287887, EPI_ISL_1287888, EPI_ISL_1287890, EPI_ISL_1287891, EPI_ISL_1287926, EPI_ISL_1287928, EPI_ISL_1287962, EPI_ISL_1288096, EPI_ISL_1288105, EPI_ISL_1288107, EPI_ISL_1288113, EPI_ISL_1288114, EPI_ISL_1288117, EPI_ISL_1288542, EPI_ISL_1288543, EPI_ISL_1288545, EPI_ISL_1288549, EPI_ISL_1288551, EPI_ISL_1288552, EPI_ISL_1288553, EPI_ISL_1288555, EPI_ISL_1288556, EPI_ISL_1288562, EPI_ISL_1288569, EPI_ISL_1288574, EPI_ISL_1288581, EPI_ISL_1288629, EPI_ISL_1288630, EPI_ISL_1288634, EPI_ISL_1288636, EPI_ISL_1288638, EPI_ISL_1288639, EPI_ISL_1288641, EPI_ISL_1288642, EPI_ISL_1288677, EPI_ISL_1288679, EPI_ISL_1288713, EPI_ISL_1288911, EPI_ISL_1288912, EPI_ISL_1288913, EPI_ISL_1288919, EPI_ISL_1288943, EPI_ISL_1288944, EPI_ISL_1289112, EPI_ISL_1289119, EPI_ISL_1289123, EPI_ISL_1289126, EPI_ISL_1289128, EPI_ISL_1289130, EPI_ISL_1289133, EPI_ISL_1289172, EPI_ISL_1289173, EPI_ISL_1369721, EPI_ISL_1369793, EPI_ISL_1369824, EPI_ISL_1369838, EPI_ISL_1369846, EPI_ISL_1369855, EPI_ISL_1369882, EPI_ISL_1369961, EPI_ISL_1369963, EPI_ISL_1369964, EPI_ISL_1369965, EPI_ISL_1369966, EPI_ISL_1369967, EPI_ISL_1369968, EPI_ISL_1369969, EPI_ISL_1369970, EPI_ISL_1369971, EPI_ISL_1369972, EPI_ISL_1369973, EPI_ISL_1369974, EPI_ISL_1369975, EPI_ISL_1369976, EPI_ISL_1369977, EPI_ISL_1369978, EPI_ISL_1369979, EPI_ISL_1369980, EPI_ISL_1369981, EPI_ISL_1369982, EPI_ISL_1369983, EPI_ISL_1369984, EPI_ISL_1369985, EPI_ISL_1369986, EPI_ISL_1369987, EPI_ISL_1369988, EPI_ISL_1369989, EPI_ISL_1369990, EPI_ISL_1369991, EPI_ISL_1369992, EPI_ISL_1369993, EPI_ISL_1369994, EPI_ISL_1369995, EPI_ISL_1369996, EPI_ISL_1369997, EPI_ISL_1369998, EPI_ISL_1369999, EPI_ISL_1370000, EPI_ISL_1370001, EPI_ISL_1370002, EPI_ISL_1370003, EPI_ISL_1370004, EPI_ISL_1370005, EPI_ISL_1370006, EPI_ISL_1370007, EPI_ISL_1370008, EPI_ISL_1370009, EPI_ISL_1370010, EPI_ISL_1370011, EPI_ISL_1370012, EPI_ISL_1370013, EPI_ISL_1370014, EPI_ISL_1370015, EPI_ISL_1370016, EPI_ISL_1370017, EPI_ISL_1370019, EPI_ISL_1370021, EPI_ISL_1370023, EPI_ISL_1370025, EPI_ISL_1370027, EPI_ISL_1370028, EPI_ISL_1370030, EPI_ISL_1370032, EPI_ISL_1370034, EPI_ISL_1370036, EPI_ISL_1370038, EPI_ISL_1370040, EPI_ISL_1370042, EPI_ISL_1370044, EPI_ISL_1370046, EPI_ISL_1370047, EPI_ISL_1370120, EPI_ISL_1370130, EPI_ISL_1370151, EPI_ISL_1370159, EPI_ISL_1370194, EPI_ISL_1370213, EPI_ISL_1370215, EPI_ISL_1370586, EPI_ISL_1370625, EPI_ISL_1370688, EPI_ISL_1370789, EPI_ISL_1370951, EPI_ISL_1370957, EPI_ISL_1370959, EPI_ISL_1370962, EPI_ISL_1370966, EPI_ISL_1370967, EPI_ISL_1370970, EPI_ISL_1370972, EPI_ISL_1370973, EPI_ISL_1370974, EPI_ISL_1370976, EPI_ISL_1370978, EPI_ISL_1370979, EPI_ISL_1370982, EPI_ISL_1370984, EPI_ISL_1370985, EPI_ISL_1370986, EPI_ISL_1370988, EPI_ISL_1370991, EPI_ISL_1370997, EPI_ISL_1370998, EPI_ISL_1370999, EPI_ISL_1371001, EPI_ISL_1371002, EPI_ISL_1371005, EPI_ISL_1371006, EPI_ISL_1371011, EPI_ISL_1371012, EPI_ISL_1371014, EPI_ISL_1371015, EPI_ISL_1371016, EPI_ISL_1371018, EPI_ISL_1371019, EPI_ISL_1371020, EPI_ISL_1371025, EPI_ISL_1371026, EPI_ISL_1371028, EPI_ISL_1371030, EPI_ISL_1371035, EPI_ISL_1371036, EPI_ISL_1371037, EPI_ISL_1371039, EPI_ISL_1371040, EPI_ISL_1371042, EPI_ISL_1371043, EPI_ISL_1371044, EPI_ISL_1371045, EPI_ISL_1371048, EPI_ISL_1371050, EPI_ISL_1371051, EPI_ISL_1371052, EPI_ISL_1371053, EPI_ISL_1371056, EPI_ISL_1371058, EPI_ISL_1371061, EPI_ISL_1371174, EPI_ISL_1371179, EPI_ISL_1371186, EPI_ISL_1371194, EPI_ISL_1371199, EPI_ISL_1371218, EPI_ISL_1371633, EPI_ISL_1371634, EPI_ISL_1371638, EPI_ISL_1371643, EPI_ISL_1371655, EPI_ISL_1371658, EPI_ISL_1371664, EPI_ISL_1371665, EPI_ISL_1371672, EPI_ISL_1371673, EPI_ISL_1455676, EPI_ISL_1455677, EPI_ISL_1455862, EPI_ISL_1455863, EPI_ISL_1455864, EPI_ISL_1455865, EPI_ISL_1455866, EPI_ISL_1455900, EPI_ISL_1456419, EPI_ISL_1456662, EPI_ISL_1456663, EPI_ISL_1456675, EPI_ISL_1456680, EPI_ISL_1456928, EPI_ISL_1456929, EPI_ISL_1456930, EPI_ISL_1456931, EPI_ISL_1456932, EPI_ISL_1456933, EPI_ISL_1456934, EPI_ISL_1456935, EPI_ISL_1456936, EPI_ISL_1456937, EPI_ISL_1456938, EPI_ISL_1456939, EPI_ISL_1456940, EPI_ISL_1456941, EPI_ISL_1456942, EPI_ISL_1456943, EPI_ISL_1456944, EPI_ISL_1456945, EPI_ISL_1456946, EPI_ISL_1456947, EPI_ISL_1456948, EPI_ISL_1456949, EPI_ISL_1456950, EPI_ISL_1456951, EPI_ISL_1456952, EPI_ISL_1456953, EPI_ISL_1456954, EPI_ISL_1456955, EPI_ISL_1456956, EPI_ISL_1456957, EPI_ISL_1456958, EPI_ISL_1456959, EPI_ISL_1456960, EPI_ISL_1456961, EPI_ISL_1456962, EPI_ISL_1456963, EPI_ISL_1456964, EPI_ISL_1456965, EPI_ISL_1456966, EPI_ISL_1456967, EPI_ISL_1456968, EPI_ISL_1456969, EPI_ISL_1456970, EPI_ISL_1456971, EPI_ISL_1456972, EPI_ISL_1456973, EPI_ISL_1456974, EPI_ISL_1456975, EPI_ISL_1456976, EPI_ISL_1456977, EPI_ISL_1456978, EPI_ISL_1456979, EPI_ISL_1456980, EPI_ISL_1456981, EPI_ISL_1456982, EPI_ISL_1456983, EPI_ISL_1456984, EPI_ISL_1456985, EPI_ISL_1456986, EPI_ISL_1456987, EPI_ISL_1456988, EPI_ISL_1456989, EPI_ISL_1456990, EPI_ISL_1456991, EPI_ISL_1456992, EPI_ISL_1456993, EPI_ISL_1456994, EPI_ISL_1456995, EPI_ISL_1456996, EPI_ISL_1456997, EPI_ISL_1456998, EPI_ISL_1457001, EPI_ISL_1457002, EPI_ISL_1457003, EPI_ISL_1457004, EPI_ISL_1457005, EPI_ISL_1457006, EPI_ISL_1457007, EPI_ISL_1457008, EPI_ISL_1457010, EPI_ISL_1457011, EPI_ISL_1457012, EPI_ISL_1457013, EPI_ISL_1457014, EPI_ISL_1457015, EPI_ISL_1457016, EPI_ISL_1457017, EPI_ISL_1457018, EPI_ISL_1457021, EPI_ISL_1457022, EPI_ISL_1457023, EPI_ISL_1457024, EPI_ISL_1457025, EPI_ISL_1457026, EPI_ISL_1457027, EPI_ISL_1457030, EPI_ISL_1457031, EPI_ISL_1457032, EPI_ISL_1457033, EPI_ISL_1457034, EPI_ISL_1457035, EPI_ISL_1457036, EPI_ISL_1457037, EPI_ISL_1457039, EPI_ISL_1457040, EPI_ISL_1457041, EPI_ISL_1457042, EPI_ISL_1457043, EPI_ISL_1457044, EPI_ISL_1457047, EPI_ISL_1457048, EPI_ISL_1457049, EPI_ISL_1457050, EPI_ISL_1457051, EPI_ISL_1457052, EPI_ISL_1457060, EPI_ISL_1457061, EPI_ISL_1457065, EPI_ISL_1457066, EPI_ISL_1457067, EPI_ISL_1457068, EPI_ISL_1457075, EPI_ISL_1457076, EPI_ISL_1457082, EPI_ISL_1457083, EPI_ISL_1457084, EPI_ISL_1457085, EPI_ISL_1457093, EPI_ISL_1457094, EPI_ISL_1457095, EPI_ISL_1457103, EPI_ISL_1457104, EPI_ISL_1457105, EPI_ISL_1457111, EPI_ISL_1457112, EPI_ISL_1457113, EPI_ISL_1457120, EPI_ISL_1457121, EPI_ISL_1457122, EPI_ISL_1457229, EPI_ISL_1457525, EPI_ISL_1457526, EPI_ISL_1457528, EPI_ISL_1457609, EPI_ISL_1521477, EPI_ISL_1596616, EPI_ISL_1596660, EPI_ISL_1596682, EPI_ISL_1596683, EPI_ISL_1596684, EPI_ISL_1596685, EPI_ISL_1596686, EPI_ISL_1596687, EPI_ISL_1596703, EPI_ISL_1596755, EPI_ISL_1596765, EPI_ISL_1596768, EPI_ISL_1596769, EPI_ISL_1596971, EPI_ISL_1596972, EPI_ISL_1596973, EPI_ISL_1596985, EPI_ISL_1597010, EPI_ISL_1597157, EPI_ISL_1597158, EPI_ISL_1597159, EPI_ISL_1597169, EPI_ISL_1597191, EPI_ISL_1597192, EPI_ISL_1597204, EPI_ISL_1597205, EPI_ISL_1597206, EPI_ISL_1597207, EPI_ISL_1705501, EPI_ISL_1705512, EPI_ISL_1705524, EPI_ISL_1705535, EPI_ISL_1705544, EPI_ISL_1705545, EPI_ISL_1705554, EPI_ISL_1705565, EPI_ISL_1792764, EPI_ISL_1792768, EPI_ISL_1961185, EPI_ISL_1961215, EPI_ISL_1961349, EPI_ISL_1961385, EPI_ISL_1961386, EPI_ISL_1961388, EPI_ISL_1961394, EPI_ISL_1961395, EPI_ISL_1961537, EPI_ISL_1961551, EPI_ISL_1961966, EPI_ISL_1962047, EPI_ISL_1962048, EPI_ISL_1962050, EPI_ISL_1962291, EPI_ISL_1962296, EPI_ISL_1962310, EPI_ISL_1962313, EPI_ISL_1962314, EPI_ISL_1962439, EPI_ISL_1962443, EPI_ISL_1962444, EPI_ISL_1962450, EPI_ISL_1962456, EPI_ISL_1962471, EPI_ISL_1962474, EPI_ISL_1962613, EPI_ISL_1962620, EPI_ISL_1962632, EPI_ISL_2092999, EPI_ISL_2093591, EPI_ISL_2093932, EPI_ISL_2093947, EPI_ISL_2093948, EPI_ISL_2093950, EPI_ISL_2093953, EPI_ISL_2093956, EPI_ISL_2093957, EPI_ISL_2093991, EPI_ISL_2093992, EPI_ISL_2093994, EPI_ISL_2093995, EPI_ISL_2093998, EPI_ISL_2093999, EPI_ISL_2094001, EPI_ISL_2094002, EPI_ISL_2094004, EPI_ISL_2094006, EPI_ISL_2094007, EPI_ISL_2094009, EPI_ISL_2094314, EPI_ISL_2094315, EPI_ISL_2217830, EPI_ISL_2217842, EPI_ISL_2219634, EPI_ISL_2220369, EPI_ISL_2476046			
see above	Dutch COVID-19 response team	National Institute for Public Health and the Environment (RIVM)	Adam Meijer; AnneMarie van den Brandt; Annelies Kroneman; Bas van der Veer; Chantal Reusken; Dennis Schmitz; Dirk Eggink; Euníce Then; Florian Zwagemaker; Harry Vennema; James Groot; Jeroen Cremer; Jolienke Hardeman; Karim Hajji; Kim Freriks; Linda van de Nes; Lisa Wijsman; Lynn Aarts; Melissa van Tuil; Robert Kohl; Ryanne Jaarsma; Sanne Bos; Sharon van den Brink; Sjoerd Kuiling; on behalf of the national COVID-19 response team
EPI_ISL_2790545, EPI_ISL_2790548	Eurofins-NMDL	Eurofins-NMDL	Alexandra Hansen; Anco Molijn; Anne Vogel; Bram Weijers; Chantal Schenker; Chinmoy Saha; Chris van Koppen; Coen de Jong; Cornel Brouwer; Diviana Lakker; Enes Sekerci; Glenn Bohms; Goyaert Roosen; Hamza Saleem; Humaira Yousaf; Iman El-Idrioui; Jelmer Eerland; Jorian Flik; Katja Plathan; Laura Weijenberg; Layonel Jacobus; Lida Cen; Lisa Dreesens; Madhavi Kharagjitsing; Marisol Williams; Mark de Jong; Marvin Ruiter; Mathijs Wielen; Maurine Leversteijn-van Hall; Melek Aras; Nienke Meijer; Ramon Koers; Rens Driel; Rosalie Van der Stap; Rousjan Amir; Roy Masius; Senay Ozturk; Simon Lansu; Thierry Janssens; Thomas van der Toorn; Tobias van der Heijde
EPI_ISL_995557	KU Leuven, Rega Institute, Clinical and Epidemiological Virology	KU Leuven, Rega Institute, Clinical and Epidemiological Virology	Bert Vanmechelen; Joan Marti-Carerras; Piet Maes; Tony Wawina-Bokalanga
EPI_ISL_1194717, EPI_ISL_1194718, EPI_ISL_1279221, EPI_ISL_1279223, EPI_ISL_1279224, EPI_ISL_1279225, EPI_ISL_1279227, EPI_ISL_1299273, EPI_ISL_1299274, EPI_ISL_1299277, EPI_ISL_1299279, EPI_ISL_1299282, EPI_ISL_1299284, EPI_ISL_1299287, EPI_ISL_1311851, EPI_ISL_1311852, EPI_ISL_1311854, EPI_ISL_1311855, EPI_ISL_1311856, EPI_ISL_1311857, EPI_ISL_1311858, EPI_ISL_1311859, EPI_ISL_1661256, EPI_ISL_1661302, EPI_ISL_1661303			
see above	Microvida	Microvida	J. Stohr; J. Verweij; Jaco Verweij; Joep Stöhr; S.D. Pas; Suzan D. Pas
EPI_ISL_1109409, EPI_ISL_1109412, EPI_ISL_1109413, EPI_ISL_1109414, EPI_ISL_1109415, EPI_ISL_1109416, EPI_ISL_1109417, EPI_ISL_1109418, EPI_ISL_1109420, EPI_ISL_1109421, EPI_ISL_1109422, EPI_ISL_1109423, EPI_ISL_1109424			
see above	Radboudumc	Radboudumc	Femke Wolters; Jordy Coolen
EPI_ISL_1660795, EPI_ISL_1660800, EPI_ISL_1712639, EPI_ISL_1712644, EPI_ISL_1712650, EPI_ISL_1712653, EPI_ISL_1827730, EPI_ISL_1827756, EPI_ISL_1827770, EPI_ISL_1827772, EPI_ISL_1827773, EPI_ISL_1827774, EPI_ISL_1827775, EPI_ISL_1827776, EPI_ISL_1827777, EPI_ISL_1827778, EPI_ISL_1827779, EPI_ISL_1827782, EPI_ISL_1827792, EPI_ISL_1827797, EPI_ISL_1827798, EPI_ISL_1827799, EPI_ISL_1827823, EPI_ISL_1827829, EPI_ISL_1827834, EPI_ISL_1827835, EPI_ISL_1827837, EPI_ISL_1827845, EPI_ISL_1827847, EPI_ISL_1827848, EPI_ISL_1841056, EPI_ISL_1841060, EPI_ISL_1841063, EPI_ISL_1841071, EPI_ISL_1841076, EPI_ISL_2230584, EPI_ISL_3346560, EPI_ISL_3346561, EPI_ISL_3346591, EPI_ISL_3346592, EPI_ISL_3346593, EPI_ISL_3346594, EPI_ISL_3346595, EPI_ISL_3385032, EPI_ISL_3385033, EPI_ISL_3385034, EPI_ISL_3385035, EPI_ISL_3385037, EPI_ISL_3385038, EPI_ISL_3385039, EPI_ISL_3385041, EPI_ISL_3385044, EPI_ISL_3385045, EPI_ISL_3385046, EPI_ISL_3385047, EPI_ISL_3385050, EPI_ISL_3385051, EPI_ISL_3385052, EPI_ISL_3385053, EPI_ISL_3385054, EPI_ISL_3385056, EPI_ISL_3385057, EPI_ISL_3385058, EPI_ISL_3385059, EPI_ISL_3385060, EPI_ISL_3385062, EPI_ISL_3385063, EPI_ISL_3385064, EPI_ISL_3385065, EPI_ISL_3385066, EPI_ISL_3385067, EPI_ISL_3385068, EPI_ISL_3486227, EPI_ISL_3486229, EPI_ISL_3486231, EPI_ISL_3486234, EPI_ISL_3491280, EPI_ISL_3491281, EPI_ISL_3491322, EPI_ISL_3491323, EPI_ISL_3532914, EPI_ISL_3535033, EPI_ISL_3535034, EPI_ISL_3535035, EPI_ISL_3535038, EPI_ISL_3535039, EPI_ISL_3535040, EPI_ISL_3535041, EPI_ISL_3535042, EPI_ISL_3535044, EPI_ISL_3535045, EPI_ISL_3535046, EPI_ISL_3535048, EPI_ISL_3535049, EPI_ISL_3535050, EPI_ISL_3535052, EPI_ISL_3535054, EPI_ISL_3535055, EPI_ISL_3535056, EPI_ISL_3535057, EPI_ISL_3535058, EPI_ISL_3535059, EPI_ISL_3535060, EPI_ISL_3535061, EPI_ISL_3535062, EPI_ISL_3535063, EPI_ISL_3535064, EPI_ISL_3535065, EPI_ISL_3535066, EPI_ISL_3535067, EPI_ISL_3535068, EPI_ISL_3535069, EPI_ISL_3535070, EPI_ISL_3535071, EPI_ISL_3535072, EPI_ISL_3535073, EPI_ISL_3535074, EPI_ISL_3535075, EPI_ISL_3535076, EPI_ISL_3535077, EPI_ISL_3535078, EPI_ISL_3535079, EPI_ISL_3535080, EPI_ISL_3535081, EPI_ISL_3535082, EPI_ISL_3535083, EPI_ISL_3535084, EPI_ISL_3535085, EPI_ISL_3535086, EPI_ISL_3535087, EPI_ISL_3535088, EPI_ISL_3535089, EPI_ISL_3535090, EPI_ISL_3535091, EPI_ISL_3535092, EPI_ISL_3535093, EPI_ISL_3535094, EPI_ISL_3535095, EPI_ISL_3535096, EPI_ISL_3535097, EPI_ISL_3535098, EPI_ISL_3535099, EPI_ISL_3535100, EPI_ISL_3535101, EPI_ISL_3535120, EPI_ISL_3535121, EPI_ISL_3535122, EPI_ISL_3535125, EPI_ISL_3535126, EPI_ISL_3535127, EPI_ISL_3535128, EPI_ISL_3535129, EPI_ISL_3535131, EPI_ISL_3535132, EPI_ISL_3535133, EPI_ISL_3535135, EPI_ISL_3535136, EPI_ISL_3535137, EPI_ISL_3535139, EPI_ISL_3535141, EPI_ISL_3535142, EPI_ISL_3535143, EPI_ISL_3535144, EPI_ISL_3535145, EPI_ISL_3535146, EPI_ISL_3535147, EPI_ISL_3535148, EPI_ISL_3535149, EPI_ISL_3535150, EPI_ISL_3535151, EPI_ISL_3535152, EPI_ISL_3535153, EPI_ISL_3535154, EPI_ISL_3535155, EPI_ISL_3535156, EPI_ISL_3535157, EPI_ISL_3535158, EPI_ISL_3535159, EPI_ISL_3535160, EPI_ISL_3535161, EPI_ISL_3535162, EPI_ISL_3535163, EPI_ISL_3535164, EPI_ISL_3535165, EPI_ISL_3535166, EPI_ISL_3535167, EPI_ISL_3535168, EPI_ISL_3535169, EPI_ISL_3535170, EPI_ISL_3535171, EPI_ISL_3535172, EPI_ISL_3535173, EPI_ISL_3535174, EPI_ISL_3535175, EPI_ISL_3535176, EPI_ISL_3535177, EPI_ISL_3535178, EPI_ISL_3535179, EPI_ISL_3535180, EPI_ISL_3535181, EPI_ISL_3535182, EPI_ISL_3535183, EPI_ISL_3535184, EPI_ISL_3535185, EPI_ISL_3535186, EPI_ISL_3535187, EPI_ISL_3535188, EPI_ISL_3535189, EPI_ISL_3535190			
see above	UMC Groningen, Clinical Virology, Department of Medical Microbiology and Infection Prevention	UMC Groningen, Clinical Virology, Department of Medical Microbiology and Infection Prevention	Alexander Friedrich; Coretta Van Leer-Buter; Erley Lizarazo-Forero; Hubert Niesters; Lilli Gard; Marjolein Knoester; Monika Fliss; Sigrid Rosema; Xuewei Zhou
